# Effect of M2-like macrophages of the injured-kidney cortex on kidney cancer progression

**DOI:** 10.1038/s41420-022-01255-3

**Published:** 2022-12-05

**Authors:** Taisuke Ishii, Imari Mimura, Koji Nagaoka, Akihiro Naito, Takehito Sugasawa, Ryohei Kuroda, Daisuke Yamada, Yasuharu Kanki, Haruki Kume, Tetsuo Ushiku, Kazuhiro Kakimi, Tetsuhiro Tanaka, Masaomi Nangaku

**Affiliations:** 1 Division of Nephrology and Endocrinology, The University of Tokyo Graduate School of Medicine, 7-3-1 Hongo, Bunkyo-ku, Tokyo, 1138655 Japan; 2 Department of Immunotherapeutics, The University of Tokyo Hospital, 7-3-1 Hongo, Bunkyo-ku, Tokyo, 1138655 Japan; 3 Division of Urology, The University of Tokyo Graduate School of Medicine, 7-3-1 Hongo, Bunkyo-ku, Tokyo, 1138655 Japan; 4 Laboratory of Clinical Examination/Sports Medicine, Division of Clinical Medicine, Faculty of Medicine, University of Tsukuba, 1-1-1 Tennodai, Tsukuba, Ibaraki 3058577 Japan; 5 Department of Pathology, The University of Tokyo Graduate School of Medicine, 7-3-1 Hongo, Bunkyo-ku, Tokyo, 1138655 Japan; 6 Department of Nephrology, Rheumatology and Endocrinology, Tohoku University Graduate School of Medicine, 1-1 Seiryo-machi, Aoba-ku, Sendai, 9808574 Japan

**Keywords:** Cancer microenvironment, Immunosurveillance

## Abstract

Chronic kidney disease (CKD) affects kidney cancer patients’ mortality. However, the underlying mechanism remains unknown. M2-like macrophages have pro-tumor functions, also exist in injured kidney, and promote kidney fibrosis. Thus, it is suspected that M2-like macrophages in injured kidney induce the pro-tumor microenvironment leading to kidney cancer progression. We found that M2-like macrophages present in the injured kidney promoted kidney cancer progression and induced resistance to anti-PD1 antibody through its pro-tumor function and inhibition of CD8^+^ T cell infiltration. RNA-seq revealed *Slc7a11* was upregulated in M2-like macrophages. Inhibition of Slc7a11 with sulfasalazine inhibited the pro-tumor function of M2-like macrophages and synergized with anti-PD1 antibody. Moreover, *SLC7A11*-positive macrophages were associated with poor prognosis among kidney cancer patients. Collectively, this study dissects the characteristic microenvironment in the injured kidney that contributed to kidney cancer progression and anti-PD1 antibody resistance. This insight offers promising combination therapy with anti-PD1 antibody and macrophage targeted therapy.

## Introduction

Cancer is more common among patients with chronic kidney disease (CKD) [1–3]. Moreover, several studies have shown that CKD increases cancer mortality [4, 5]. Specifically, CKD is associated with an increased risk of cancer death among kidney cancer patients [6]. The association between CKD and cancer mortality remained evident after adjusting for chemotherapy and patients’ factors, including performance status [7]. These facts indicate that CKD contributes to cancer progression. However, to date, few studies have assessed the biological mechanism by which CKD worsens cancer progression.

Macrophages are a well-known major component of the innate immune system and display several phenotypes in response to each microenvironment. In the injured kidney, macrophages (especially M2-like macrophages) are related to fibrosis in several organs, including the kidneys [8]. Tumor-associated macrophages (TAMs) are a major component of the tumor microenvironment (TME) [9]. TAMs contain two phenotypes: anti-tumor (M1-like) and pro-tumor (M2-like) [10]. There are many known differences between M1-like and M2-like TAMs, including differences in transcription factors, cytokine production and function [11, 12]. Recent studies showed that susceptibility to ferroptosis, which is mainly caused by oxidative stress, and the expression level of ferroptosis-related genes were different between M1-like and M2-like TAMs [13, 14]. M2-like TAMs enhance tumor progression in various types of cancer [10]. Although previous studies reported various results, M2-like TAMs generally release immunosuppressive cytokines, transforming growth factor-beta, and vascular endothelial growth factor (VEGF), leading to tumor progression and drug resistance [15–17]. In kidney cancer, M2-like TAMs similarly promote cancer progression through angiogenesis and stimulation of tumor proliferation [18]. Additionally, in clear cell renal cell carcinoma (ccRCC), certain types of macrophages correlated to the low prevalence of tumor-infiltrating T cells and poor prognosis [19].

Although immune checkpoint inhibitors (ICIs) have recently become the standard therapy for several types of cancer, including ccRCC and non ccRCC [20], resistance to ICIs remains a major clinical problem [21, 22]. Therefore, the development of new therapeutic strategies is urgently needed for various types of cancer [23, 24]. Generally, the presence of M2-like macrophages is recognized as a factor that reduces the efficacy of ICIs [21, 25]. High expression of myeloid signature has been associated with poor efficacy among metastatic ccRCC patients who received atezolizumab monotherapy, which is one of the ICIs [26]. Recently, macrophage-targeted therapies have been investigated to complement the effect of ICIs [10, 21]. Collectively, these results suggest that myeloid cells, especially M2-like TAMs, would decrease the efficacy of ICIs leading to poor prognosis. However, whether M2-like macrophages in the injured kidney affect the efficacy of ICI therapy in kidney cancer patients remains unclear.

Macrophages have important roles both in kidney fibrosis and kidney cancer progression. Therefore, we hypothesized that M2-like macrophages in injured kidneys contribute to kidney cancer progression leading to poor prognosis and resistance to ICI therapy among kidney cancer patients with CKD. To test this hypothesis, we evaluated murine kidney cancer progression in injured kidneys and assessed the relationship between M2-like macrophages in injured kidneys and kidney cancer progression. Furthermore, we assessed whether these macrophages affected ICI efficacy and explored the genetic features of M2-like macrophages in this microenvironment. This study would provide a basis for the development of promising combination therapy of ICI and macrophage-targeting therapy, which modulates macrophage function, for kidney cancer patients with CKD.

## Results

### Kidney cancer is more progressive in injured kidneys with F4/80^low^Ly6C^low^ macrophage infiltration than in healthy kidneys

**Fig. 1:**
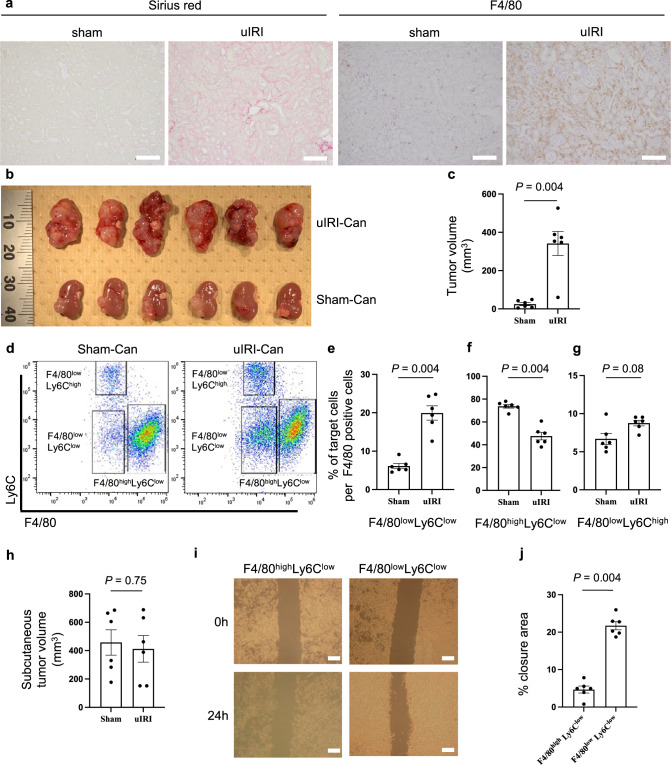

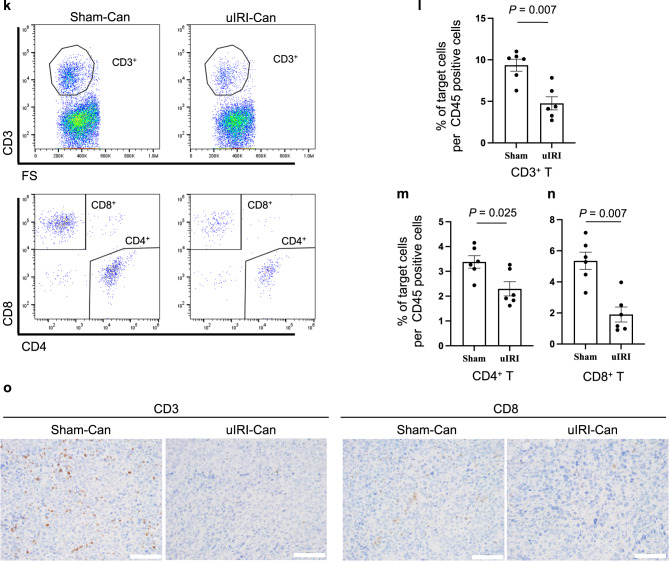
Kidney cancer progression in the injured kidney is enhanced with an increase in F4/80^low^Ly6C^low^ M2-like macrophages and decrease in tumor-infiltrating T cells. **a** uIRI was conducted for 30 min, and mice were euthanized 14 days after uIRI. Kidney fibrosis and macrophage accumulation in the renal cortex was assessed. Representative Sirius red staining (left) and immunohistochemical analysis of F4/80 expression (right) in the kidney cortex are shown. Scale bars: 100 μm. **b**–**g** RenCa cells were inoculated into subcapsular kidney 14 days after uIRI (uIRI-Can) or sham-operation (Sham-Can). Mice were euthanized 20 days after inoculation. **b** Picture of uIRI-Can and Sham-Can is shown. **c** The tumor volume of uIRI-Can and Sham-Can is shown. (*n* = 6 per group; two-sided Mann–Whitney *U* test). **d** F4/80^low^Ly6C^low^ cells, F4/80^high^Ly6C^low^ cells, and F4/80^low^Ly6C^high^ cells were detected by flow cytometry. One plot from each group (Sham-Can (left) and uIRI-Can (right)) is shown. Percentages of F4/80^low^Ly6C^low^ cells (**e**), F4/80^high^Ly6C^low^ cells (**f**), and F4/80^low^Ly6C^high^ cells (**g**) among F4/80^+^ cells are shown. (*n* = 6 per group; two-sided Mann–Whitney *U* test). **h** RenCa cells were subcutaneously injected 14 days after uIRI or sham operation, and mice were euthanized on day 20 after tumor injection. The tumor volume of each group is shown. (*n* = 6 per group; two-sided Mann–Whitney *U* test). **i**, **j** RenCa cells were co-cultured with F4/80^low^Ly6C^low^ or F4/80^high^Ly6C^low^ macrophages in Transwell systems for 24 h after creating a gap in the confluent monolayer of RenCa cells, and the degree of gap closure was evaluated. (*n* = 6 per group). **i** Representative picture of each group is shown at time 0 and 24 h after starting co-culture. Scale bars: 300 μm. **j** The quantification of the percentage of the closure area is shown. (two-sided Mann–Whitney *U* test). **k** CD3^+^, CD4^+^, and CD8^+^ tumor-infiltrating T cells were detected by flow cytometry. One plot from each group (Sham-Can (left) and uIRI-Can (right)) is shown. **l**–**n** CD3^+^, CD4^+^, and CD8^+^ tumor-infiltrating T cell proportion in CD45^+^ cells were quantitatively evaluated in uIRI-Can and Sham-Can, as determined by flow cytometer. (*n* = 6 per group; two-sided Mann–Whitney *U* test). **o** Representative immunohistochemistry images of uIRI-Can and Sham-Can stained for CD3 (left) and CD8 (right) are shown. Scales bar, 100 μm. uIRI unilateral ischemia-reperfusion injury, uIRI-Can kidney cancer inoculated into the kidney subcapsule after unilateral IRI, Sham-Can kidney cancer inoculated into the kidney subcapsule after sham operation.

Kidney fibrosis was induced after unilateral ischemia-reperfusion injury (uIRI), a well-established acute kidney injury (AKI) to CKD model in vivo [27–30]. Sirius red staining indicated that kidney fibrosis developed on day 14 after uIRI, and accumulation of F4/80^+^ macrophages was detected in the injured kidney cortex (Fig. 1a). Next, we inoculated RenCa cells, which is a BALB/c derived murine kidney cancer cell line, into renal subcapsule 14 days after uIRI to examine tumor growth and tumor-infiltrating macrophages in the tumor tissue. Kidney cancer in the uIRI kidney (uIRI-Can) progressed more aggressively than that in the sham-treated kidney (Sham-Can) (Fig. 1b, c). In uIRI-Can, the proportion of F4/80^low^Ly6C^low^ macrophages in F4/80^+^Gr1^−^ cells was higher (Fig. 1d, e), and the proportion of F4/80^high^Ly6C^low^ macrophages was lower (Fig. 1f), compared to that in Sham-Can. However, F4/80^low^Ly6C^high^ macrophage proportions were not significantly different between the two groups (Fig. 1g).

We also evaluated kidney cancer progression in the injured kidney in another well-known AKI to CKD model using aristolochic acid (AA) [31, 32]. We confirmed that AA administration induced kidney fibrosis and F4/80^+^ macrophages accumulation (Extended Data Fig. 1a). Similar to the uIRI model, kidney cancer progressed more aggressively in AA kidneys than in sham kidneys (Extended Data Fig. 1b, c), and the F4/80^low^Ly6C^low^ macrophage proportion was higher in the AA group than in the control group (Extended Data Fig. 1d). F4/80^high^Ly6C^low^ and F4/80^low^Ly6C^high^ macrophage proportions were not different between the two groups (Extended Data Fig. 1e, f). Since Ly6C^high^ cells were theoretically recognized as pro-inflammatory cells [33, 34] and the proportion of these cells was not different in the uIRI and AA model, hereafter we compared the two groups: F4/80^low^Ly6C^low^ and F4/80^high^Ly6C^low^ populations.

Since uremia is associated with an immunosuppressive state among end-stage renal disease patients [35], we assessed the kidney function in uIRI model and evaluated the systemic effect of uIRI on tumor progression. We collected blood samples from uIRI treated mice 14 days after surgery and measured plasma creatinine and plasma urea nitrogen levels. Both plasma creatinine and plasma urea nitrogen levels were not significantly different between the uIRI-treated and sham-operated mice group (Extended Data Fig. 2a, b), indicating that the systemic filtration function of kidneys was not impaired due to the unilateral kidney damage, consistent with the previous studies [30]. To examine the systemic function of injured kidneys, we subcutaneously inoculated RenCa cells. There was no significant difference in tumor growth between the uIRI and sham groups (Fig. 1h and Extended Data Fig. 2c). These results indicated that the local microenvironment of injured kidneys with accumulating macrophages mainly affected kidney cancer progression, but not systemic factors, including uremia.

### F4/80^low^Ly6C^low^ macrophage expresses M2-like phenotype and directly promotes kidney cancer progression

To characterize F4/80^low^Ly6C^low^ macrophages, we sorted F4/80^low^Ly6C^low^ macrophages from uIRI-Can using cell sorter and compared the expression of several signature genes of classical M1/M2 macrophages between F4/80^low^Ly6C^low^ and F4/80^high^Ly6C^low^ macrophages. F4/80^low^Ly6C^low^ macrophages expressed higher levels of *Arg1* and *Vegfa*, which are known as M2 macrophage markers (Extended Data Fig. 3a, b). The expression of *Il10*, an M2 macrophage marker, was not different between the F4/80^low^Ly6C^low^ and F4/80^high^Ly6C^low^ macrophages (Extended Data Fig. 3c). F4/80^low^Ly6C^low^ macrophages also expressed higher levels of *Il1β* and *Il6*, which are M1 macrophage markers; however, *Tnfa* expression level was not different between the two groups (Extended Data Fig. 3d–f). Thus, F4/80^low^Ly6C^low^ macrophages shared classical M1 and M2 marker gene expressions.

To assess the F4/80^low^Ly6C^low^ macrophage phenotype, we conducted a scratch healing assay. F4/80^low^Ly6C^low^ or F4/80^high^Ly6C^low^ macrophages were co-cultured with RenCa cells after creating a gap in the confluent layer of RenCa cells (time 0) using Transwell systems. Gap closure area was evaluated by comparing the gap area at time 0 to that at 24 h after starting co-culture. The percentage of closure area was 4 times higher in RenCa cells co-cultured with F4/80^low^Ly6C^low^ macrophages than in those co-cultured with F4/80^high^Ly6C^low^ macrophages (Fig. 1i, j).

This result showed that F4/80^low^Ly6C^low^ macrophages promoted RenCa cell migration, although F4/80^low^Ly6C^low^ co-expressed signature genes of M1 and M2 macrophages. Recent studies showed that pro-tumor macrophages shared classical M1 and M2 marker gene expressions [36–38]. Collectively, F4/80^low^Ly6C^low^ macrophages were characterized as pro-tumor M2-like macrophages because F4/80^low^Ly6C^low^ macrophages enhanced the migration ability of RenCa cells.

### Tumor-infiltrating T cell population decreases in the case of kidney cancer in an injured kidney

Next, we compared the proportion of tumor-infiltrating T cells in uIRI-Can with that in Sham-Can. Flow cytometry analyses showed that the proportion of intra-tumor CD3^+^ (Fig. 1k, l), CD4^+^ (Fig. 1m), and CD8^+^ T cells (Fig. 1n) in CD45^+^ cells was lower in uIRI-Can than in Sham-Can. Consistently, immunohistochemistry showed that the number of tumor-infiltrating CD3 and CD8 T cells was lower in uIRI-Can than in Sham-Can (Fig. 1o). Similarly, the proportion of CD3^+^ (Extended Data Fig. 4a), CD4^+^ (Extended Data Fig. 4b), and CD8^+^ T cells (Extended Data Fig. 4c) was lower in kidney cancer inoculated on AA-treated kidneys than that on PBS treated kidneys.

We also evaluated regulatory T cell (Treg) infiltration, the cytokine producing T cell function, and the proportion of exhausted CD8 T cells. The CD4^+^ FoxP3^+^ Treg proportion in CD4^+^ T cells was not different between uIRI-Can and Sham-Can (Extended Data Fig. 5a). Regarding T cell function, interferon-γ (IFNγ)^+^ CD8^+^ T cell and tumor necrotic factor-α (TNFα)^+^ CD8^+^ T cell proportion in CD8^+^ T cells was not different between uIRI-Can and Sham-Can (Extended Data Fig. 5b, c). The percentage of PD1^+^Tim3^+^CD8^+^ T cells in CD8^+^ T cells was not different between the two groups (Extended Data Fig. 5d). These results indicated that there were no significant differences in the status and function of tumor-infiltrating CD8^+^ T cells and Treg infiltration; however, tumor-infiltration of T cells was hampered in the injured kidney.

### Adoptive transfer of M2-like macrophages promotes kidney cancer progression

**Fig. 2:**
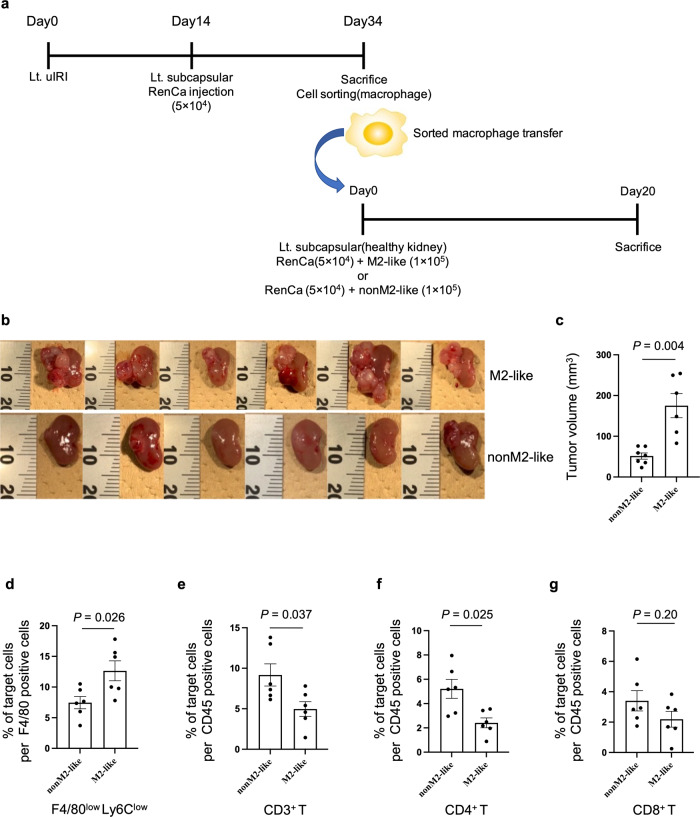
Adoptive transfer of M2-like macrophages from kidney cancer reproduces the immunosuppressive tumor microenvironment. F4/80^low^Ly6C^low^ (M2-like) or F4/80^high^Ly6C^low^ (nonM2-like) macrophages, which were sorted from uIRI-Can, were co-injected with RenCa cells into healthy kidney subcapsule. Tumor progression was evaluated 20 days after co-injection. **a** The protocol of this experiment is shown. **b**, **c** Tumor volume of M2-like macrophage co-injected kidney cancer and nonM2-like macrophage co-injected kidney cancer is shown. (*n* = 6 per group; two-sided Mann–Whitney *U* test). **d** Intra-tumor F4/80^low^Ly6C^low^ macrophage proportion in F4/80^+^ cells was quantitatively evaluated in M2-like macrophage co-injected tumor and non-M2-like macrophage co-injected tumor, as determined by flow cytometer. (*n* = 6 per group; two-sided Mann–Whitney *U* test). Tumor-infiltrating CD3^+^ T cells (**e**), CD4^+^ T cells (**f**), and CD8^+^ T cells (**g**) proportion in CD45^+^ cells were quantitatively evaluated in M2-like macrophage co-injected tumor and non-M2-like macrophage co-injected tumor, as determined by flow cytometer. (*n* = 6 per group; two-sided Mann–Whitney *U* test). Lt. left side, uIRI unilateral ischemia-reperfusion injury.

To examine whether M2-like macrophages promoted kidney cancer progression in vivo, we sorted F4/80^low^Ly6C^low^ M2-like macrophages or F4/80^high^Ly6C^low^ non-M2-like macrophages from uIRI-Can and adoptively transferred these macrophages into RenCa cells co-injected with F4/80^low^Ly6C^low^ or F4/80^high^Ly6C^low^ macrophages into the subcapsular kidney of healthy Balb/c mice together with 5 × 10^4^ RenCa cells (Fig. 2a).

RenCa cells showed increased progression following co-injection with F4/80^low^Ly6C^low^ macrophages compared to that with F4/80^high^Ly6C^low^ macrophages (Fig. 2b, c). In F4/80^low^Ly6C^low^ macrophage co-injected tumor, the F4/80^low^Ly6C^low^ macrophage proportion was higher (Fig. 2d) and the proportions of tumor-infiltrating CD3^+^ (Fig. 2e) and CD4^+^ T cells (Fig. 2f) were lower, compared to those in F4/80^high^Ly6C^low^ macrophage co-injected tumor. The intra-tumor CD8^+^ T cell proportion was not statistically different between the two groups (Fig. 2g). These results indicated that M2-like macrophage reproduced the immunosuppressive TME and suppressed the infiltration of T cells into the tumor.

### F4/80^low^Ly6C^low^ macrophage acts as M2-like macrophage in the injured kidney cortex

Since previous studies showed that inhibition of Ly6C^low^ M2-like macrophage infiltration after uIRI led to a reduction in renal fibrosis [29, 39], we assessed the role of F4/80^low^Ly6C^low^ macrophage in the injured kidney cortex by evaluating the effect of macrophage depletion on renal fibrosis using clodronate liposomes (CL).

At first, we confirmed the F4/80^low^Ly6C^low^ macrophage accumulation in the uIRI kidney cortex. The F4/80^low^Ly6C^low^ macrophage proportion increased in the kidney cortex 14 days after uIRI compared to that in the sham kidney cortex (Extended Data Fig. 6a). Similar to F4/80^low^Ly6C^low^ macrophages in tumor tissue, F4/80^low^Ly6C^low^ macrophages sorted from the uIRI kidney cortex highly expressed *Arg1* and *Vegfa* compared to F4/80^high^Ly6C^low^ macrophages (Extended Data Fig. 6b).

**Fig. 3:**
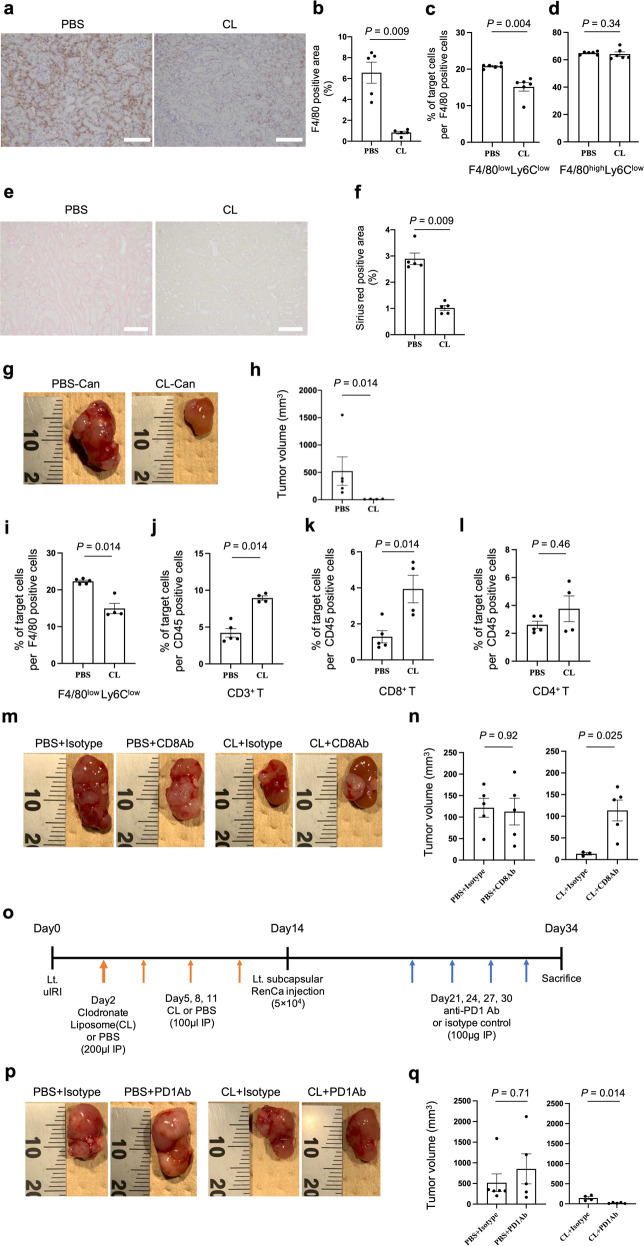
F4/80^low^Ly6C^low^ M2-like macrophages in the injured kidney cortex promote kidney cancer progression and modulate ICI therapy efficacy via increased intra-tumor M2-like macrophages and decreased tumor-infiltrating CD8 T cells. Clodronate liposomes (CL) or PBS was administered every 2 days starting the day after uIRI and mice were euthanized 14 days after uIRI. **a** Representative immunohistochemical analysis of F4/80 expression in PBS-treated uIRI-kidney cortex (left) and CL-treated uIRI-kidney cortex (right) are shown. Scale bars: 100 μm. **b** Corresponding graph indicates the percentage of F4/80 positive area. (*n* = 5 per group; two-sided Mann–Whitney *U* test). **c**, **d** F4/80^low^Ly6C^low^, and F4/80^high^Ly6C^low^ macrophage proportion in F4/80^+^ cells were quantitatively evaluated in PBS-treated uIRI-kidney cortex and CL-treated uIRI-kidney cortex, as determined by flow cytometer. (*n* = 6 per group; two-sided Mann–Whitney *U* test). **e** Representative Sirius red staining of PBS-treated uIRI-kidney cortex (left) and CL-treated uIRI-kidney cortex (right) are shown. Scale bars: 100 μm. **f** Corresponding graph indicates the percentage of Sirius red positive area. (*n* = 5 per group; two-sided Mann–Whitney *U* test). **g**–**k** RenCa cells were inoculated into uIRI kidney subcapsule which was treated with CL (CL-Can) or PBS (PBS-Can) after uIRI. **g** Representative photos of each tumor are shown. **h** The tumor volume of CL-Can and PBS-Can is shown. (*n* = 5 per PBS-Can, *n* = 4 per CL-Can; two-sided Mann–Whitney *U* test). **i** Intra-tumor F4/80^low^Ly6C^low^ macrophage proportion in F4/80^+^ cells was quantitatively evaluated in CL-Can and PBS-Can, as determined by flow cytometer. (*n* = 5 per PBS-Can, *n* = 4 per CL-Can; two-sided Mann–Whitney *U* test). Tumor-infiltrating CD3^+^ T cells (**j**), CD8^+^ T cells (**k**), and CD4^+^ T cells (**l**) proportion in CD45^+^ cells were quantitatively evaluated in CL-Can and PBS-Can, as determined by flow cytometer. (*n* = 5 per PBS-Can, *n* = 4 per CL-Can; two-sided Mann–Whitney *U* test). Anti-CD8 or isotype control was administered after RenCa cell inoculation into uIRI kidney subcapsule which was treated with CL or PBS after uIRI. **m** Representative photos of each tumor are shown. **n** Tumor volume is shown; anti-CD8 antibody or isotype control administration to kidney cancer in PBS-treated uIRI kidney (left) (*n* = 5 per group), anti-CD8 antibody or isotype control administration to kidney cancer in CL-treated uIRI kidney (right) (*n* = 3 per isotype control group, *n* = 5 per anti-CD8 antibody group; two-sided Mann–Whitney *U* test). **o**–**q** Mouse anti-PD1 antibody or isotype control was administered after RenCa cell inoculation into uIRI kidney subcapsule which was treated with CL or PBS after uIRI. **o** The protocol of this experiment is shown. **p** Representative photos of each tumor are shown. **q** Tumor volume of anti-PD1 antibody or isotype control administered kidney cancer is shown; anti-PD1 antibody or isotype control administration to kidney cancer in PBS-treated uIRI kidney (left) (*n* = 6 per isotype control group, *n* = 5 per anti-PD1 antibody group), anti-PD1 antibody or isotype control administration to kidney cancer in CL-treated uIRI kidney (right) (*n* = 4 per isotype control group, *n* = 5 per anti-PD1 antibody group; two-sided Mann–Whitney *U* test). Lt. left side, uIRI unilateral ischemia-reperfusion injury, CL clodronate liposomes, Ab antibody.

CL administration after uIRI eliminated F4/80 positive macrophages that infiltrated in the injured kidney cortex (Fig. 3a, b) and reduced F4/80^low^Ly6C^low^ proportion (Fig. 3c). Contrarily, the F4/80^high^Ly6C^low^ proportion did not decrease (Fig. 3d). Moreover, CL administration attenuated kidney fibrosis in the uIRI kidney cortex compared to PBS treatment (Fig. 3e, f). These results indicated that, in our model, F4/80^low^Ly6C^low^ macrophages acted as M2-like macrophages also in the injured kidney cortex because F4/80^low^Ly6C^low^ macrophage depletion led to attenuation of kidney fibrosis.

### Injured kidney cortex promotes kidney cancer progression via increased M2-like macrophage infiltration in tumor tissue that inhibited the infiltration of CD8 T cells into the tumor

To evaluate the relationship between M2-like macrophage in the injured kidney cortex and progression of uIRI-Can, we inoculated RenCa cells into subcapsular uIRI kidney treated with CL (CL-Can) or PBS (PBS-Can). Cancer progression was inhibited in CL-Can compared to that in PBS-Can (Fig. 3g, h). F4/80^low^Ly6C^low^ macrophage proportion was decreased in CL-Can compared to that in PBS-Can, together with increased tumor-infiltrating CD3^+^ and CD8^+^ T cell proportion in CL-Can (Fig. 3i–k). However, the CD4^+^ T cell proportion was not statistically different between the two groups (Fig. 3l). These results indicated that M2-like macrophage depletion from the uIRI kidney cortex improved the immunosuppressive TME and resumed T cell infiltration into the tumor.

Next, we evaluated the contribution of CD8^+^ or CD4^+^ T cell infiltration in uIRI-Can by depleting these cells using anti-CD8 antibody or anti-CD4 antibody to CL-Can or PBS-Can (Extended Data Fig. 7a). RenCa cell progression was not affected by the anti-CD8 antibody (Fig. 3m, n) although CD8^+^ T cells were effectively depleted from tumor tissue using the anti-CD8 antibody (Extended Data Fig. 7b). As expected, macrophage elimination by CL inhibited the progression of RenCa cells. However, CD8^+^ T cell depletion in CL-Can abrogated the anti-tumor effect of F4/80^low^Ly6C^low^ macrophage elimination (Fig. 3m, n). The anti-CD4 antibody did not affect tumor progression in both CL-Can and PBS-Can (Extended Data Fig. 7c, d) although the anti-CD4 antibody depleted CD4^+^ T cells from tumor tissue (Extended Data Fig. 7e). Based on these results, we showed that M2-like macrophages in the injured kidney cortex expanded the M2-like macrophage population in uIRI-Can. Additionally, these M2-like macrophages inhibited CD8^+^ T cell infiltration into the kidney cancer tissue and caused kidney cancer progression.

### M2-like macrophage diminishes the effect of ICIs

Recently, ICIs have been the standard therapy for kidney cancer patients [40]. However, resistance to ICI remains a major problem. Furthermore, M2-like macrophage infiltration and CD8^+^ T cell exclusion have been recognized as TME promoting factors that reduce ICI efficacy [25]. To compare the efficacy of ICI in kidney cancer patients with CKD to those without CKD, we assessed 49 kidney cancer patients who received ICI therapy in our hospital. Among them, 36 patients were diagnosed with CKD at the time of ICI initiation (Supplementary Table 1). In the Cox regression analysis, after adjusting for age (<65 vs. 65≦ years old) and the International Metastatic Renal Cell Carcinoma Database Consortium (IMDC) score (0 or 1 vs. 2), which is an established prognostic score for metastatic kidney cancer patients [41], CKD was significantly associated with short time to ICI failure (hazard ratio = 3.82, 95% confidence interval: 1.10–13.3; *p* = 0.035, Supplementary Table 2). This result indicated that the TME in kidney cancer which developed in injured kidneys would modulate the ICI efficacy. To evaluate whether M2-like macrophages in uIRI-Can modulate ICI efficacy, we assessed the effect of anti-PD1 treatment on CL-Can and PBS-Can (Fig. 3o). Although anti-PD1 antibodies could not inhibit tumor growth in PBS-Can, the anti-PD1 antibodies inhibited tumor progression in CL-Can where M2-like macrophages were depleted from injured kidney before tumor inoculation (Fig. 3p, q). These results indicated that M2-like macrophages present in the injured kidney cortex encouraged kidney cancer progression and caused resistance to ICI therapy.

### Transcriptome analysis of M2-like macrophages in injured kidney cortex and kidney cancer

**Fig. 4:**
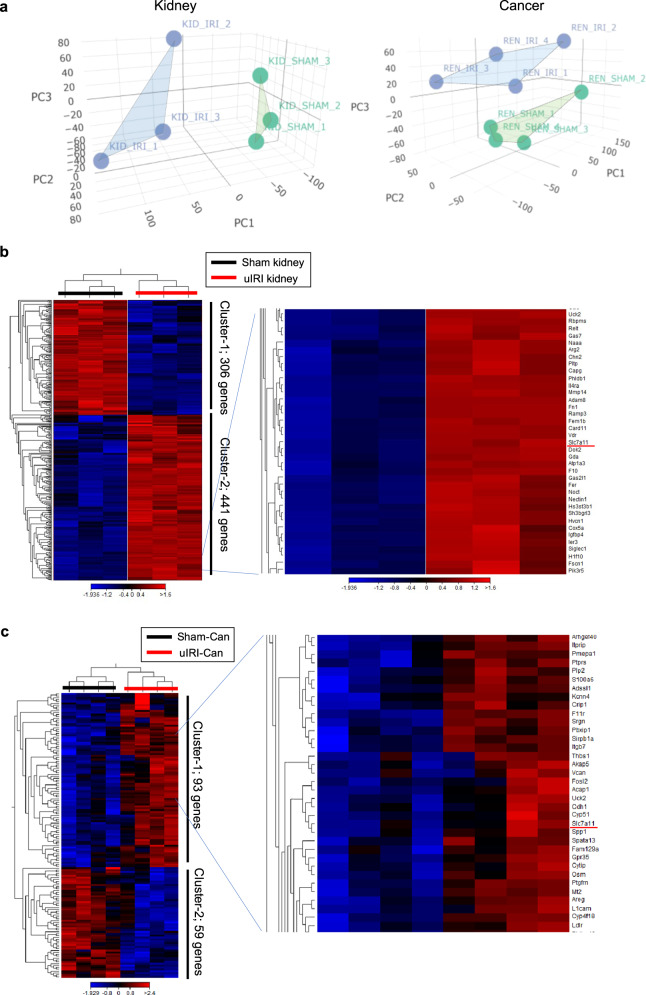

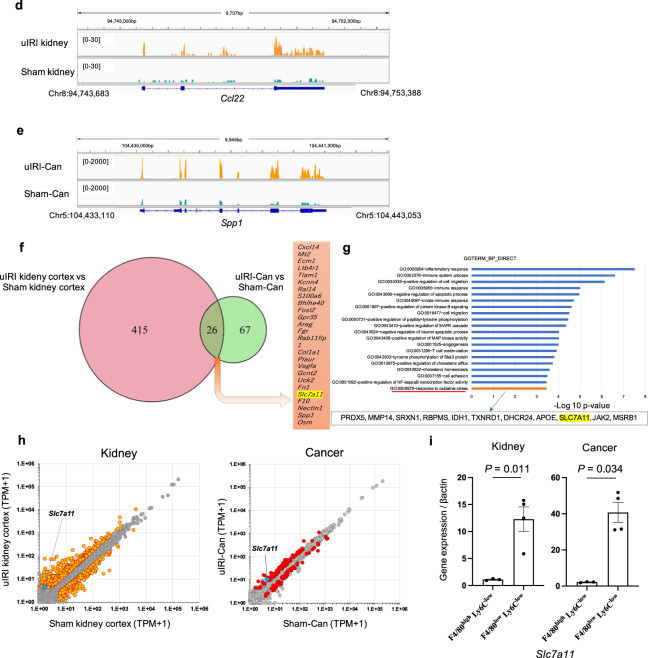
M2-like macrophages express higher levels of *Slc7a11* than non-M2-like macrophages. RNA-seq was conducted for four different types of Ly6C^low^ macrophages; uIRI kidney cortex, sham kidney cortex, uIRI-Can, and Sham-Can. (*n* = 3 per each kidney sample, *n* = 4 per each tumor sample). **a** Principal component analysis (PCA) shows different expression patterns between uIRI-treated samples and Sham-treated samples among Ly6C^low^ macrophages in the kidney (left) and Ly6C^low^ macrophages in cancer (right); blue color represents uIRI-treated samples, green color represents Sham-treated samples. **b**, **c** Heatmap shows differentially expressed genes. **b** 441 genes were upregulated and 306 genes were downregulated in Ly6C^low^ macrophages sorted from the uIRI kidney cortex compared to those from the sham kidney cortex with FDR < 0.001 and log fold change (log FC) more than 2. **c** 93 genes were upregulated and 59 genes were downregulated in Ly6C^low^ macrophages sorted from uIRI-Can compared to those from Sham-Can with FDR < 0.05. Representative gene tracks of RNA-seq signals around *Ccl22* in kidney samples (**d**), and *Spp1* in tumor samples (**e**). RNA-seq signals are visualized using Integrative Genomics Viewer (Version 2.11.3) (http://software.broadinstitute.org/software/igv/). **f** 26 genes were commonly upregulated in Ly6C^low^ macrophages sorted from the uIRI kidney cortex and uIRI-Can. **g** Top 20 Gene Ontology (GO) terms enriched among upregulated genes in uIRI kidney included the response to oxidative stress. **h** Scatter plots show the locus of *Slc7a11* among differentially expressed genes in kidney sample (left), and in tumor sample (right). **i** In qPCR, *Slc7a11* expression was upregulated in F4/80^low^Ly6C^low^ macrophages compared to F4/80^high^Ly6C^low^ macrophages both in uIRI kidney cortex (left) and uIRI-Can (right). (*n* = 3 per each F4/80^high^Ly6C^low^ macrophage group, *n* = 4 per each F4/80^low^Ly6C^low^ macrophage group; two-sided Mann–Whitney *U* test). uIRI unilateral ischemia-reperfusion injury, uIRI-Can kidney cancer inoculated into the kidney subcapsule after unilateral IRI, Sham-Can kidney cancer inoculated into the kidney subcapsule after sham operation.

Next, we conducted RNA-Sequencing (RNA-Seq) of sorted Ly6C^low^ macrophages to identify gene characteristics of M2-like macrophages that were commonly detected both in uIRI kidney cortex and uIRI-Can. All normalized gene expressions detected in these four groups (uIRI kidney cortex, sham kidney cortex, uIRI-Can, and Sham-Can) are summarized in Supplementary Data 1. Principal component analysis (PCA) showed that the expression pattern of Ly6C^low^ macrophages derived from the uIRI-treated group was separated from that of Ly6C^low^ macrophages derived from the Sham-treated group both in kidney and cancer samples (Fig. 4a). In Ly6C^low^ macrophages sorted from the uIRI kidney, 441 genes were upregulated and 306 genes were downregulated compared to those sorted from the sham kidney (Fig. 4b). Ninety-three genes were highly expressed and 59 genes were poorly expressed in Ly6C^low^ macrophages sorted from uIRI-Can compared to those from Sham-Can (Fig. 4c). Consistent with a previous study [29], *Ccl22* was highly expressed in uIRI kidney cortex samples and was identified within differentially expressed genes (DEGs) in uIRI kidney samples (Fig. 4d). Moreover, the expression of *Spp1*, which is considered a pro-tumor macrophage marker [36, 42], was higher in uIRI-Can samples than in Sham-Can samples, and it was contained in DEGs of uIRI-Can samples (Fig. 4e). Additionally, we confirmed that the expression of *Vegfa* was upregulated both in uIRI kidney and uIRI-Can samples using qPCR (Extended Data Fig. 8a, b). The list of DEGs is provided in Supplementary Data 2. Among DEGs that were highly expressed in uIRI-treated samples, 26 genes were commonly upregulated in the uIRI-Can and uIRI kidney cortex (Fig. 4f). We focused on the 26 commonly upregulated genes to evaluate the genetic features of M2-like macrophages both in the injured kidney cortex and kidney cancer. Although susceptibility to ferroptosis, which is mainly induced by oxidative stress, in cancer cells has been recognized to contribute to cancer progression and resistance to several therapies [43], recent single-cell RNA-seq analysis showed that ferroptosis-related genes were expressed in some clusters of macrophages [14]. Another study showed that susceptibility to ferroptosis was related to macrophage subtypes; M1-like or M2-like [13]. A previous study showed that IL4-induced M2 macrophages expressed *Slc7a11*, a ferroptosis-related gene which codes cystine/glutamate antiporter (xCT) [43], and *Slc7a11* expression was increased in the wound healing tissue in vivo. However, the function of *Slc7a11* positive macrophages remains unclear, especially in the TME. In this study, differentially upregulated genes in the uIRI kidney were involved in response to oxidative stress (Fig. 4g). Additionally, *Slc7a11* was included in 26 commonly upregulated genes. Therefore, we hypothesized that *Slc7a11* might be a characteristic gene of M2-like macrophages (Fig. 4b, c, h). qPCR confirmed that *Slc7a11* was highly expressed in Ly6C^low^ macrophages derived from uIRI kidney and uIRI-Can (Extended Data Fig. 8c).

Considering that increased F4/80^low^Ly6c^low^ macrophage infiltration was detected in uIRI tumors and kidneys compared to in sham groups (Extended Data Fig. 8d), F4/80^low^Ly6c^low^ macrophages might be responsible for the increased expression of *Slc7a11* in uIRI kidney and uIRI-Can. qPCR confirmed that *Slc7a11* expression was upregulated in F4/80^low^Ly6C^low^ macrophages compared to that in F4/80^high^Ly6C^low^ macrophages in both uIRI kidney and uIRI-Can (Fig. 4i).

### Sulfasalazine improves immune checkpoint inhibitor efficacy by changing M2-like macrophage function

**Fig. 5:**
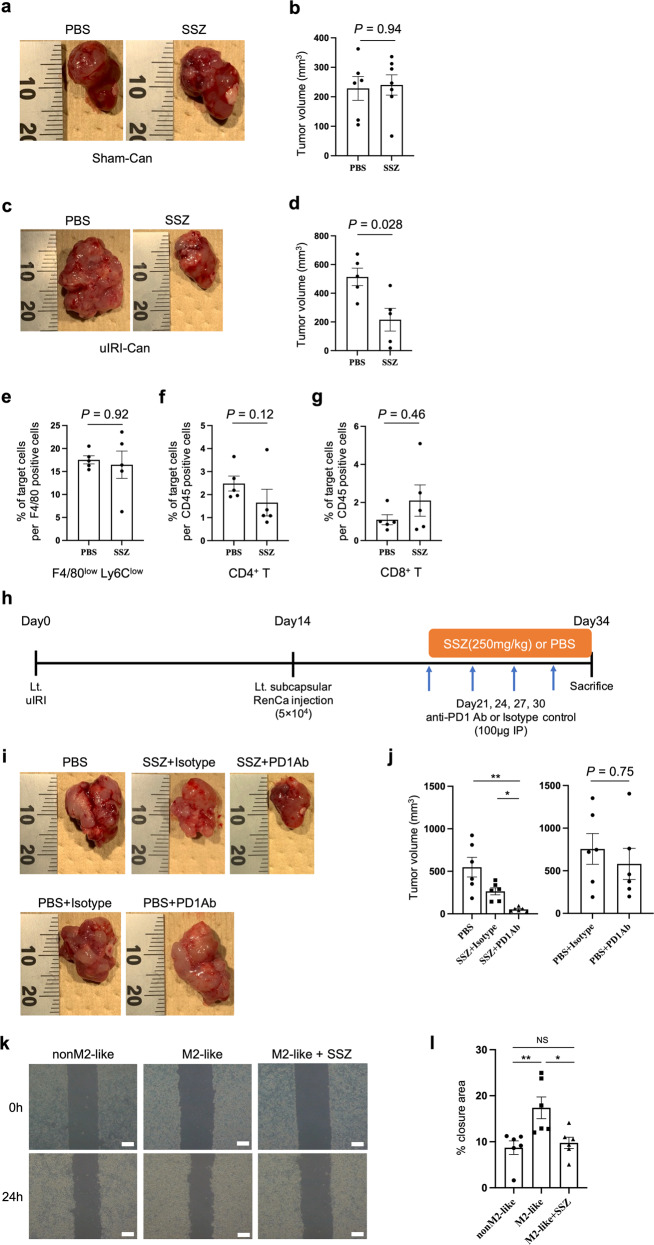
Sulfasalazine improves anti-PD1 antibody resistance in kidney cancer inoculated into uIRI kidney subcapsule via changing direct pro-tumor function of M2-like macrophages. **a**, **b** Sulfasalazine (SSZ) or PBS was administered every day starting 7 days after tumor inoculation into sham-operated kidney subcapsule. **a** Representative photos of each tumor are shown. **b** The tumor volume of each group is shown. (*n* = 6 per group; two-sided Mann–Whitney *U* test).　**c**, **d** SSZ or PBS was administered every day starting 7 days after tumor inoculation into uIRI-operated kidney subcapsule. **c** Representative photos of each tumor are shown. **d** The tumor volume of each group is shown. (*n* = 5 per group; two-sided Mann–Whitney *U* test). **e** Intra-tumor F4/80^low^Ly6C^low^ macrophage proportion in F4/80^+^ cells was quantitatively evaluated, as determined by flow cytometer. (*n* = 5 per group; two-sided Mann–Whitney *U* test). Tumor-infiltrating CD4^+^ T cells (**f**), and CD8^+^ T cells (**g**) proportion in CD45^+^ cells were quantitatively evaluated, as determined by flow cytometer. (*n* = 5 per group; two-sided Mann–Whitney *U* test). **h**–**j** SSZ or PBS was daily administered, and mouse anti-PD1 antibody or isotype control was administered every 2 days starting 7 days after RenCa cell inoculation into uIRI kidney subcapsule. **h** The protocol of this experiment is shown. **i** Representative photos of each tumor are shown. **j** The tumor volume of each group is shown. (*n* = 6 per group). *p < 0.05, **p < 0.01. Statistical analysis was performed using Kruskal–Wallis test followed by a post hoc Dann–Bonferroni multiple comparisons test or two-sided Mann–Whitney *U* test. **k**, **l** RenCa cells were co-cultured with F4/80^low^Ly6C^low^(M2-like), SSZ-treated F4/80^low^Ly6C^low^(M2-like+SSZ), or F4/80^high^Ly6C^low^(nonM2-like) macrophages in Transwell systems for 24 h after creating a gap in confluent monolayer of RenCa cells, and the degree of gap closure was evaluated. (*n* = 6 per group). **k** Representative picture of each group is shown at time 0 and 24 h after starting co-culture. Scale bars: 300 μm. **l** The quantification of the percentage of closure area is shown. Black circle represents non-M2-like group, black square represents M2-like group, and black triangle represents M2-like + SSZ group. NS not significant; *p < 0.05, **p < 0.01 (Kruskal–Wallis test followed by a post hoc Dann–Bonferroni multiple comparisons test). Lt. left side, uIRI unilateral ischemia-reperfusion injury, CL clodronate liposome, Ab antibody, uIRI-Can kidney cancer inoculated into the kidney subcapsule after unilateral IRI, Sham-Can kidney cancer inoculated into the kidney subcapsule after sham operation, SSZ sulfasalazine.

Sulfasalazine (SSZ), which is usually used for ulcerative colitis and rheumatoid arthritis [44, 45], is one of the currently available inhibitors for xCT [46–48]. Although SSZ shows anti-tumor activity in some tumors [47, 49, 50], SSZ did not affect RenCa cell viability and did not have cell toxicity for RenCa cells (Extended Data Fig. 9a, b). To confirm the SSZ efficacy in RenCa cells, we intraperitoneally injected SSZ in mice with Sham-Can. Consistently, SSZ did not affect the progression of tumor inoculated into subcapsule of healthy kidney (Fig. 5a, b). Next, we evaluated the efficacy of SSZ on M2-like macrophages. First, we confirmed that SSZ inhibited cystine uptake ability of xCT on F4/80^low^Ly6C^low^ macrophages but it did not affect the cystine uptake of F4/80^high^Ly6C^low^ non-M2-like macrophages (Extended Data Fig. 9c). Since the difference in the cystine uptake inhibition by SSZ between F4/80^low^Ly6C^low^ and F4/80^high^Ly6C^low^ macrophages reflects the difference in xCT expression between F4/80^low^Ly6C^low^ and F4/80^high^Ly6C^low^ macrophages, this result supported our RNA-seq result that xCT expression was upregulated in F4/80^low^Ly6C^low^ M2-like macrophages compared to in F4/80^high^Ly6C^low^ non-M2-like macrophages. Next, we assessed SSZ toxicity in F4/80^low^Ly6C^low^ macrophages. SSZ did not affect the cell viability of F4/80^low^Ly6C^low^ macrophages and did not have cell toxicity for these cells (Extended Data Fig. 9d, e). However, intraperitoneal injection of SSZ in mice with uIRI-Can inhibited tumor progression (Fig. 5c, d). SSZ administration did not affect the proportion of F4/80^low^Ly6C^low^ macrophages and CD4^+^ and CD8^+^ T cells (Fig. 5e–g). Since previous studies showed that xCT-mediated glutamate release to the TME recruited Tregs in some cancers [51, 52], we also evaluated the proportion of Treg. In our model, SSZ did not affect the proportion of Treg in uIRI-Can (Extended Data Fig. 9f).

Additionally, the uIRI-Can mice were treated with a combination of anti-PD1 and SSZ (Fig. 5h), which augmented the anti-tumor activity of the anti-PD1 antibody (Fig. 5i, j). These results indicated that SSZ inhibited tumor progression in M2-like macrophage-rich TME although SSZ did not affect the survival and polarization of M2-like macrophages and CD8^+^ T cell infiltration.

To evaluate the effect of SSZ on M2-like macrophage function, we co-cultured RenCa cells and F4/80^low^Ly6C^low^ macrophages pretreated with or without SSZ and performed a scratch healing test (Extended Data Fig. 9g). Compared to F4/80^high^Ly6c^low^ macrophages, F4/80^low^Ly6C^low^ macrophages promoted RenCa cell migration. In contrast, SSZ treatment abolished the induction of RenCa cell migration by F4/80^low^Ly6C^low^ macrophages (Fig. 5k, l). These results suggested that SSZ modified the pro-tumor activities of M2-like macrophages that might augment the efficacy of the anti-PD1 antibody, and the mechanism of this pro-tumor function was different from that of inhibiting effector T cell infiltration.

### SLC7A11-high macrophages are detected in human cancers

**Fig. 6:**
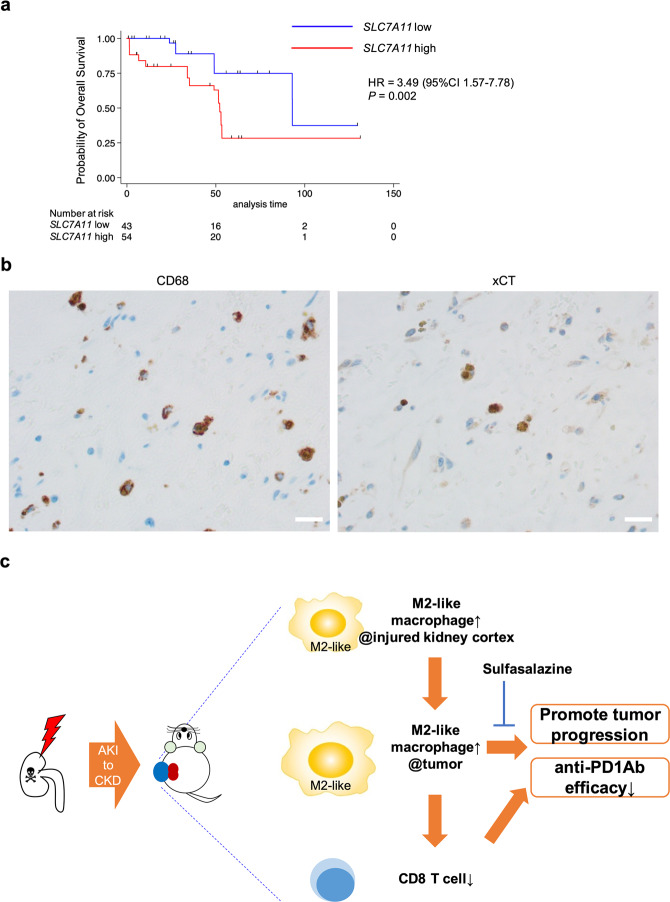
*SLC7A11* positive macrophages exist in human kidney cancer and are associated with poor prognosis. **a** Kaplan–Meier plot for comparing the overall survival between kidney cancer patients with high expression of *SLC7A11* and kidney cancer patients with low expression of *SLC7A11*; blue line indicates *PTPRC*-high and *SLC7A11*-low patients, and red line indicates *PTPRC*-high and *SLC7A11*-high patients. The overall survival of kidney cancer patients with high expression of *SLC7A11* was worse than patients with low expression of *SLC7A11*. Statistical analysis was performed using the two-sided log-rank test. HR was calculated using univariate Cox regression analysis. **b** Representative immunohistochemical analysis of xCT-positive CD68^+^ macrophages in kidney cancer patients. Scale bars: 20 μm. **c** Schematic summary of the main results. Through AKI to CKD process, M2-like macrophages were accumulated in the injured kidney cortex. These M2-like macrophages in the injured kidney expanded the M2-like macrophage population in the kidney cancer tissue, leading to the progression of kidney cancer and diminished the efficacy of the anti-PD1 antibody via at least two distinct mechanisms—a direct mechanism to tumor progression and inhibition of CD8 T cell infiltration into the tumor. SSZ modulated the direct pro-tumor of M2-like macrophages and improved the anti-tumor activity and susceptibility to anti-PD1 antibody therapy. HR hazard ratio, CI confidence interval, AKI acute kidney injury, CKD chronic kidney disease, SSZ sulfasalazine.

To determine whether *SLC7A11*-positive macrophages exist in human cancers, we evaluated *SLC7A11-expressing* macrophages using a publicly available single-cell RNA-seq dataset consisting of tumor-infiltrating myeloid cells in pan-cancer [36]. In kidney cancer, *SLC7A11*-positive macrophages were mainly detected in *GPNMB* positive or *FN1* positive macrophage sub-clusters (Extended Data Fig. 10a, b). Previous studies reported that GPNMB production by macrophages promoted tumor progression and metastasis [53, 54]. Additionally, *FN1* positive macrophages are associated with poor prognosis in kidney cancer patients [36], and *Fn1* was detected among our 26 commonly upregulated genes. Using clinical data from the TCGA dataset, we evaluated the association between *SLC7A11* expression in myeloid cells and overall survival. Kidney cancer patients with *SLC7A11*-positive myeloid cells had a poor prognosis (Fig. 6a). We confirmed that xCT-positive macrophages existed in human kidney cancer tissue using immunohistochemistry (Fig. 6b). On the contrary, *SLC7A11*-positive non-myeloid cells were not associated with prognosis among kidney cancer patients (Extended Data Fig. 10c). Also, based on our preliminaly result, xCT expression was not detected in human kidney cancer cells. Moreover, using the same single-cell RNA-seq dataset, we evaluated *SLC7A11*-positive macrophages in other cancer types with *SPP1*-positive macrophages, a poor prognostic factor based on previous studies [36, 42], because *Spp1* was also detected in our 26 commonly upregulated genes. *SLC7A11*-positive macrophages were identified in lung, colon, ovarian, and pancreatic cancer patients, and these macrophages were mainly detected among *SPP1* positive macrophage sub-clusters (Extended Data Fig. 10d, e). These results showed that *SLC7A11*-positive macrophages were also detected in pro-tumor M2-like macrophage subclusters in human cancers, and they were associated with poor prognosis especially among kidney cancer patients.

## Discussion

The TME has been recognized as a promising therapeutic target because it can modulate cancer growth, invasion, metastasis, and response to several therapies [55]. TAMs are one of the main components in TME; specifically, M2-like TAMs are associated with poor prognosis and resistance to ICI in several types of cancer owing to their pro-tumor and immunosuppressive function [10, 40, 56]. Previous studies evaluated the contribution of macrophages present in healthy organs to tumor progression. A recent study reported that normal tissue-resident macrophages surrounding cancer cells supported tumor progression by promoting cancer invasion and recruiting Treg in early non-small cell lung cancer (NSCLC) [57]. However, few studies evaluated the feature of macrophages present in the TME of cancer that develops in injured organs. Regarding kidneys, previous studies have shown that M2-like macrophages have important roles in fibrosis and tumor progression. Ly6C^low^ macrophages accumulated in injured kidneys after uIRI and promoted kidney fibrosis through myofibroblast activation [29], and Ly6C^low^ macrophages present in tumor tissue promoted tumor progression in vivo [58, 59]. However, the relationship between M2-like macrophages in injured kidneys and kidney cancer progression remains to be evaluated. Here, we demonstrated that preexisting M2-like macrophages in the injured kidney cortex have an important role in expanding the M2-like macrophage population in kidney cancer, promoting kidney cancer progression and resistance to ICI. The immunosuppressive microenvironment with M2-like macrophages was already established in the injured kidney cortex and facilitated kidney cancer progression (Fig. 6c).

Pharmacological inhibition of xCT using SSZ reduced the growth of lymphoma cells and has been shown to inhibit tumor growth and metastasis in several types of cancer [44, 46, 60]. Additionally, xCT inhibition using SSZ in glioblastoma cells caused an increase in reactive oxygen species leading to cell death, which was recognized as ferroptosis [61]. However, in our study, SSZ did not affect the progression of kidney cancer without M2-like macrophages, and xCT inhibition of M2-like macrophages using SSZ did not cause an increase in cell death or reduce the percentage of M2-like macrophages in tumor tissues. Contrarily, SSZ suppressed the pro-tumor function of M2-like macrophages although SSZ did not improve the recruitment of CD8^+^ T cells into tumor tissue. These results suggested that xCT in M2-like macrophages contributed to the progression of kidney cancer and SSZ did not deplete M2-like macrophages, and M2-like macrophages had two distinct pro-tumor activities: promoting the migration ability of cancer cells and inhibiting CD8 T cell infiltration, and the underlying mechanisms of these two functions were different. A previous study showed systemic xCT knockout did not affect T cell infiltration into grafted tumors and T cell proliferation [62]. Based on previous reports, it was considered that M2-like macrophages contributed to the abnormal structure of tumor neovessels leading to inhibition of T cell infiltration although M2-like macrophages had complicated functions [63, 64]. Therefore, future studies are needed to clarify the precise mechanism underlying M2-like macrophage-mediated inhibition of CD8^+^ T cell infiltration to develop effective macrophage-targeted therapy which enhances the efficacy of immunotherapy.

Although macrophage depletion therapy is expected to be effective against cancer [38, 40, 65], recent data showed that whole macrophage depletion was insufficient to inhibit cancer progression due to heterogeneity and diverse functions of macrophages. The anti-tumor effect of CSF-1R inhibition, which leads to inhibition of macrophage recruitment in cancer tissue, showed limited anti-tumor activity as a monotherapy [66]. Therefore, recently, more specific strategies for pro-tumor macrophages or reprogramming macrophage function to attenuate pro-tumor or immunosuppressive ability are investigated [64]. One preclinical study showed that modulating M2-like macrophage function was a promising cancer therapy and it improved the efficacy of radiation therapy in the glioblastoma model [67]. Our study suggested that SSZ would be useful to overcome the resistance to ICI by modulating the pro-tumor function of *Slc7a11*-positive M2-like macrophages. Future studies should evaluate the efficacy of SSZ on ICI-resistant kidney cancer with background injured kidneys.

While SSZ inhibits xCT-mediated cystine uptake, all current available xCT inhibitors, including SSZ, had low target specificity against xCT [68]. Therefore, although SSZ could change the M2-like macrophage function, whether this effect was caused only by xCT inhibition remains unclear. Further studies which use myeloid cell-specific *Slc7a11* knockout mice will be needed to confirm the effect of *Slc7a11* on M2-like macrophage function. However, this is the first study that showed SSZ could affect the M2-like macrophage function and improve susceptibility to ICI therapy.

Moreover, we showed that *SLC7A11*-positive macrophages were detected not only in kidney cancer but also in lung, colon, ovarian, and pancreatic cancer. Furthermore, these macrophages were mainly found in *SPP1*-positive macrophage subcluster associated with poor prognosis based on previous studies [36, 42], and *Spp1* was upregulated in M2-like macrophages in both injured kidneys and kidney cancer in our study, similar to *Slc7a11*. In these organs, M2-like macrophages are also recognized to contribute to promoting organ failure and cancer progression. Idiopathic pulmonary fibrosis (IPF) is a risk factor for the incidence of NSCLC [69], and the complication of IPF is a poor prognostic factor among NSCLC patients [70]. M2-like macrophages had a profibrotic role in the bleomycin-induced pulmonary fibrosis model [71]. Additionally, M2-like macrophages promoted the progression of NSCLC [72]. In chronic pancreatitis, M2-like macrophages promoted fibrosis and chronic inflammation [73]. VEGF secreting M2-like macrophages enhanced angiogenesis, invasion, and metastasis of pancreatic cancer [74]. Moreover, M2-like macrophages contributed to cancer progression in several cancers, including colon [75] and ovarian cancer [76]. Based on these reports and our results, M2-like macrophages present in damaged organs might promote cancer progression in these organs. Furthermore, *Slc7a11*-positive M2-like macrophages would be a promising therapeutic target especially for cancers that develop in chronically injured organs.

In conclusion, M2-like macrophages present in the injured kidney cortex increased the M2-like macrophage population in kidney cancer. It led to kidney cancer progression and diminished the effect of the anti-PD1 antibody therapy via at least two distinct mechanisms: a direct mechanism to tumor progression and inhibition of CD8^+^ T cell infiltration into the tumor. While ICIs are already one of the key therapies for metastatic kidney cancer, recently, adjuvant pembrolizumab monotherapy after nephrectomy showed efficacy for improving disease-free survival [77]. Although the importance of ICIs in kidney cancer treatment continues to grow, the resistance to ICIs remains one of the most important issues. M2-like macrophages, which preexist around the tumor, especially in injured kidneys, potentially mediate the resistance to ICI therapy in kidney cancer. Our results would contribute to predicting the ICI therapy efficacy and developing specific combination therapy for kidney cancer patients with injured kidney.

## Methods

### Cell culture and reagents

RenCa cells, a BALB/c derived murine kidney cancer cell line, were purchased from Cell Lines Service and maintained in RPMI1640 medium (Cell Lines Service, Eppelheim, Germany) at 37°C with 5% CO_2_. Sulfasalazine (SSZ) used in this study was obtained from Sigma-Aldrich (St. Louis, Missouri, USA; S0883).

### Unilateral ischemia-reperfusion injury

Wild-type BALB/c male mice (7–8 weeks of age) were used for all experiments. All mice were purchased from Nippon Bio-Supp. Center (Tokyo, Japan). Mice were anesthetized by intraperitoneal injection of medetomidine (0.3 mg/kg), butorphanol (5 mg/kg), and midazolam (4 mg/kg). Body temperature was monitored using a rectal probe and maintained between 36 ± 0.5 °C with a heating pad (Neuroscience Inc, Osceola, WI, USA). Renal uIRI was performed by clamping the left-side renal pedicle for 30 min. The clamp was released, and the wound was sutured after the restoration of blood flow was visually observed. Sham-operated mice underwent the same procedure without clamping renal pedicles. Fourteen days after reperfusion, mice were euthanized or received RenCa cells inoculation. Mice were randomly assigned to each group when they were purchased. Blinding was not performed.

### Aristolochic acid administration

AA (A5512, Sigma-Aldrich, St. Louis, Missouri, USA) was dissolved in PBS and administered intraperitoneally to the mice (5 mg/ kg). Fourteen days after AA administration, mice were euthanized or received RenCa cells inoculation.

### RenCa transplantation

RenCa cells (5 × 10^4^) were inoculated into left-side kidney subcapsule 14 days after uIRI or AA administration or sham operation [78, 79]. Mice were anesthetized in the same way as described above. The left-side kidney was exposed through the incision site and a needle was inserted under the renal capsule to inject the RenCa cell suspension. Next, the wound was sutured. To detect 2 SD difference at 80% power with two-sided 5% significance level, 4–6 mice in each group were used. For subcutaneous RenCa cell transplantation, RenCa cell suspension (5 × 10^5^) was inoculated under skin 14 days after uIRI or sham operation [80]. Tumor size was measured using a caliper and calculated using the following formula: volume = (length × (width)^2^)/2.

To evaluate the efficacy of anti-PD1 antibody in our model, 100 μg of mouse anti-PD1 antibodies (clone RMP1-14, BioXcell, Lebanon, NH, USA) or 100 μg of rat IgG2a isotype control (clone 2A3, BioXcell, Lebanon, NH, USA) were intraperitoneally injected four times every 2 days starting 7 days after tumor inoculation [81]. To evaluate the efficacy of SSZ on RenCa cell progression, SSZ dissolved in PBS (250 mg/kg) or PBS were intraperitoneally injected every day starting 7 days after tumor inoculation into sham-operated kidney subcapsule. To modulate the effect of M2-like macrophages on cancer progression and anti-PD1 antibody resistance, SSZ or PBS were intraperitoneally injected every day starting 7 days after tumor inoculation into uIRI-operated kidney subcapsule with or without the anti-PD1 antibodies. Mice were randomly divideded into each treatment group when they were purchased. Blinding was not performed.

### Cell preparation and flow cytometry

Tumors were harvested and dissociated using Tissue and Tumor Dissociation Reagent (661563, BD Bioscience, Franklin Lakes, NJ, USA) according to the manufacturer’s instructions. Kidney cortex samples were cut into pieces and incubated in HEPES containing RPMI-1640 (R5886, Sigma-Aldrich, St. Louis, Missouri, USA) supplemented with 0.2% collagenase (FUJIFILM Wako Pure Chemical Corporation, Osaka, Japan) and 2 KU/ml DNase I (Sigma-Aldrich, St. Louis, Missouri, USA) for 40 min at 37 °C. Next, single-cell suspensions were prepared using a 70 μm cell strainer. To eliminate dead cells, the single-cell suspensions were stained with Zombie Yellow (BioLegend, San Diego, CA, USA). The preparations were pretreated with Fc Block (anti-CD16/32 clone 2.4G2; BioXcell, Lebanon, NH, USA) and stained with antibodies for cell-surface antigens for 30 min on ice. For intracellular cytokine staining, cells were stimulated with phorbol 12-myristate 13-acetate (PMA) 10 ng/ml (Sigma-Aldrich, St. Louis, Missouri, USA) and ionomycin 1 μM (Sigma-Aldrich, St. Louis, Missouri, USA) in the presence of brefeldin A 10 μg/ml (Sigma-Aldrich, St. Louis, Missouri, USA) for 4 h at 37 °C [82]. After marking dead cells using the Zombie Yellow (BioLegend, San Diego, CA, USA), cells were blocked with an anti-CD16/32 antibody (clone 2.4G2; BioXcell, Lebanon, NH, USA) and stained with indicated antibodies for cell-surface antigens. According to manufacturers’ instructions, cells were stained with intracellular antibodies after fixation and permeabilization using Fixation Buffer and Intracellular Staining Perm Wash Buffer (BioLegend, San Diego, CA, USA). For FoxP3 staining, following fixation and permeabilization using True-Nuclear Transcription Factor Buffer Set (424401, BioLegend, San Diego, CA, USA) as per the manufacturers’ instructions, cells were stained with anti-FoxP3 antibody (clone MF-14; BioLegend, San Diego, CA, USA).

Data were acquired using a Gallios^TM^ flow cytometer (Beckman-Coulter, Brea, CA, USA) and analyzed using the FlowJo software V.10.6.2 (BD Biosciences, Franklin Lakes, NJ, USA). The anti-mouse antibodies used in this study are listed as follows and were obtained from BioLegend (San Diego, CA, USA): FITC-conjugated anti-CD3 (clone 145-2C11), FITC-conjugated anti-CD45 (clone 30-F11), FITC-conjugated anti-CD8b (clone YTS156.7.7), PE-conjugated anti-CD45 (clone 30-F11), PE-conjugated anti-FoxP3 (clone MF-14), PE-conjugated anti-TNFα (clone MF-14), PE-CF594-conjugated anti-F4/80 (clone T45-2342), PerCP/ Cy5.5-conjugated anti-CD11b (clone M1/7), PerCP/ Cy5.5-conjugated anti-CD4 (clone GK1.5), PE/Cy7-conjugated anti-Ly6C (clone HK1.4), PE/Cy7-conjugated anti-PD1 (clone RMP1-30), PE/Cy7-conjugated anti-IFNγ (clone XMG12), APC-conjugated anti-F4/80 (clone BM8), APC-conjugated anti-Tim3 (clone RMT3-23), APC-conjugated anti-CD4 (clone GK1.5), APC-conjugated anti-CD3 (clone 145-2C11), APC/Cy7-conjugated anti-CD8a (clone 53-6.7), APC/Cy7-conjugated anti-CD45 (clone 30-F11), Pacific Blue-conjugated anti-Gr1 (clone RB6-8C5), Pacific Blue-conjugated anti-CD45 (clone 30-F11), and Pacific Blue-conjugated anti-CD8a (clone 53-6.7). PE-CF594-conjugated anti-B220 antibody (clone RA3-6B2) was obtained from BD Horizon (Franklin Lakes, NJ, USA).

### Cell sorting

Tumors were harvested from donor mice and dissociated using Tissue and Tumor Dissociation Reagent (661563, BD Bioscience, Franklin Lakes, NJ, USA) according to the manufacturer’s instructions. After passing through a 70 μm cell strainer, the single-cell suspensions were pretreated with Fc Block (anti-CD16/32 clone 2.4G2; BioXcell, Lebanon, NH, USA) and stained with FITC-conjugated anti-CD45 (clone 30-F11, BioLegend), PerCP/ Cy5.5-conjugated anti-CD11b (clone M1/70, BioLegend, San Diego, CA, USA), PE/Cy7-conjugated anti-Ly6C (clone HK1.4, BioLegend, San Diego, CA, USA), APC-conjugated anti-F4/80 (clone BM8, BioLegend, San Diego, CA, USA), Pacific Blue-conjugated anti-Gr1 (clone RB6-8C5, BioLegend, San Diego, CA, USA) antibodies, and propidium iodide (P4864, Sigma-Aldrich, St. Louis, Missouri, USA). Each target cell was sorted using SH800S (SONY, Tokyo, Japan). The data were analyzed using the FlowJo software V.10.6.2 (BD Biosciences, Franklin Lakes, NJ, USA).

### Adoptive transfer of sorted macrophages

Tumor cells were inoculated into kidney subcapsule after uIRI. Fourteen days later, tumors were harvested and single-cell suspensions were made through a 70 μm cell strainer. CD45^+^ CD11b^+^ Gr1^−^ F4/80^high^ Ly6C^low^ cells and CD45^+^ CD11b^+^ Gr1^−^ F4/80^low^ Ly6C^low^ cells were sorted using SH800S (SONY, Tokyo, Japan). F4/80^low^Ly6C^low^ or F4/80^high^Ly6C^low^ macrophages (1 × 10^5^) were co-injected with RenCa cells (5 × 10^4^) on healthy kidney subcapsule. Tumor progression was evaluated 20 days after co-injection.

### Macrophage depletion from the injured kidney

CL (CLD-8909, Encapsula NanoSciences, Brentwood, TN, USA) or PBS were intraperitoneally injected at a dose of 200 μl the day after uIRI, followed by three doses of 100 μl every 2 days [29]. Fourteen days after uIRI, mice were euthanized or received RenCa cells inoculation.

### Lymphocyte depletion

Mouse anti-CD8α antibody (clone 53-6.7, BioXcell, Lebanon, NH, USA) or anti-CD4 antibody (clone GK1.5, BioXcell, Lebanon, NH, USA) were intraperitoneally injected at a dose of 200 μg three times every 6 days starting the day after tumor inoculation [58, 83]. Equal amounts of IgG isotype antibodies (BioXcell, Lebanon, NH, USA) were intraperitoneally injected as a control.

### Immunohistochemistry

Kidney samples were fixed in Mildform 10 N (133–10311; Wako, Osaka, Japan) and embedded in paraffin. Embedded tissues were sectioned at 3 μm for immunohistochemistry. Tissue sections were dewaxed and rehydrated using gradient ethanol. For F4/80 staining, the endogenous peroxidase activity was quenched with 0.3% H_2_O_2_ in PBS for 20 min at room temperature. These sections were incubated with Protein Block Serum-Free (Dako by agent Technologies, Santa Clara, CA, USA) for 10 min at room temperature to block non-specific antibody binding. Sections were incubated with rat anti-F4/80 antibody (1/400, MCA497GA, Bio-Rad Laboratories Inc., Hercules, CA, USA) overnight at 4 °C. After washing with PBS, these slides were incubated with Histofine Simple Stain MAX PO (Nichirei Biosciences Inc., Tokyo, Japan) for 30 min at room temperature. The color was developed using ImmPACT DAB (Vector Laboratories Inc., Burlingame, CA, USA) followed by counterstaining with hematoxylin solution. Finally, slides were counterstained with hematoxylin and dehydrated. For CD3 and CD8 staining, after protein blocking, heat-induced antigen retrieval was performed using an autoclave in sodium citrate buffer (pH 6.0) at 120 °C for 20 min. Sections were incubated with rabbit anti-CD3 antibody (1/1000, ab16669, abcam, Cambridge, UK) or anti-CD8 antibody (1/300, ab209775, abcam, Cambridge, UK) overnight at 4 °C. Next, the endogenous peroxidase quenching was performed using 0.3% H_2_O_2_. Sections were incubated with biotinylated goat anti-rabbit IgG as a secondary antibody (1/1000, BA-1000, Vector Laboratories Inc., Burlingame, CA, USA) for 60 min and subsequently incubated with R.T.U. VECTASTAIN Elite ABC Reagent (Vector Laboratories Inc., Burlingame, CA, USA) for 30 min. The sections were then incubated with ImmPACT DAB (Vector Laboratories Inc., Burlingame, CA, USA) followed by counterstaining with hematoxylin solution. To evaluate tissue fibrosis, collagen 1 and collagen 3 were stained with Picrosirius red stain (Polysciences, Inc., Warrington, PA, USA).

For human samples, hematoxylin-eosin staining and immunohistochemical staining was performed using the Ventana Benchmark XT autostainer (Ventana Medical Systems, Tucson, AZ, USA). The primary antibodies used in this study were as follows: monoclonal mouse anti-CD68 antibody (1/200, M087601-2, Agilent, Santa Clara, CA, USA) and polyclonal rabbit anti-xCT antibody (1/1000, NB300-318, Novus Biologicals, Centennial, CO, USA).

### Plasma creatinine and plasma urea nitrogen level measurement

The blood sample was obtained 14 days after uIRI. Plasma creatinine and urea nitrogen level was measured using an enzymatic method following each manufacturers’ protocol (Wako Pure Chemical Industries, Osaka, Japan).

### RNA extraction and quantitative real-time PCR

Total mRNA was isolated using RNAiso Plus (Takara Bio, Shiga, Japan), and reverse transcribed using PrimeScript RT Master Mix (Takara Bio, Shiga, Japan) according to the manufacturer’s instructions. The cDNA was used for real-time quantitative PCR (qPCR), using THUNDERBIRD qPCR Mix (Toyobo, Tokyo, Japan) and a CFX96 Real Time System (Bio-Rad). β-actin was used to normalize the mRNA expression levels. The results were analyzed by the ⊿⊿Ct method. The primer sequences are listed in Supplementary Table 3.

### RNA-seq library preparation and sequencing

Using cell sorter, Ly6C^low^ macrophages were isolated from uIRI-Can, Sham-Can, uIRI kidney cortex, and sham kidney cortex. Since RNA-Seq could not be performed due to the extremely low amount of total RNA when F4/80^low^Ly6C^low^ and F4/80^high^Ly6C^low^ macrophages were collected separately, the samples were sorted together as Ly6C^low^ macrophages from each tissue; uIRI kidney cortex, sham kidney cortex, uIRI-Can, and Sham-Can. Total RNA was isolated using the RNeasy Micro Kit (74004, Qiagen, Hilden, Germany) following manufacturers’ instructions. The integrity of the RNA was checked using Agilent RNA 6000 Nano Kit (Cat# 5067-1511; Agilent) on the Bioanalyzer (Agilent). The RNA Integrity Number in all samples was 6.0 to 9.4; thus, it was considered that RNAs have partial fragmentation and total RNA sequencing is suitable in this study. Using ~120 ng of the RNAs for each sample, the libraries were created using NEBNext Ultra II RNA Library Prep Kit for Illumina and NEBNext rRNA Depletion Kit v2 (New England Biolabs, Cat# E7770S and E7400L) according to the manufacturer’s instructions, and the final PCR was performed as 15 thermal cycles. Concentrations and size distributions of the libraries were measured using an Agilent DNA 7500 kit (Cat#5067-1506; Agilent) on Bioanalyzer. All samples were passed for analysis on NGS equipment.

The libraries were pooled and the concentrations were adjusted to 1 nM. The pooled libraries were subjected to denaturation and neutralization. Subsequently, the libraries were diluted to 1.8 pM and then applied for an NGS run on the NextSeq 500 System (Illumina, San Diego, CA, USA) with NextSeq500/550 v2.5 (75 Cycles) Kits (Illumina, Cat#20024906). The sequencing was performed with paired-end reads of 36 bases. After the sequencing run, FASTQ files were exported and basic information of the NGS run data was checked on CLC Genomics Workbench 21.0.3 software (CLC, QIAGEN). In the results, the PHRED-score as a quality score of the reads over 20 was confirmed for 99% of all reads, indicating the success of the data acquisition in the NGS run. The read number was ~29–34 million per sample as paired-end reads.

### RNA-seq data analysis

The quality of FASTQ files was evaluated using FastQC (http://www.bioinformatics.babraham.ac.uk/projects/fastqc) version 0.11.8 and trimmed using Trimmomatic PE version 0.38 [84] with “ILLUMINACLIP:adaptor_sequence.fa:2:30:7:1:true LEADING:3 TRAILING:3 SLIDINGWINDOW:4:15 CROP:120 MINLEN:36” parameters. The trimmed FASTQ files were aligned to the mouse reference genome mm10 using Hisat2 version 2.1.0 [85] with a “--dta” option. SAM files were sorted and converted into BAM files using Samtools version 1.9 [86]. Gene expression was quantified in transcripts per kilobase million (TPM) using StringTie version 1.3.4d [87] with an “-e” parameter; the GTF file was downloaded from GENCODE release M20 (https://www.gencodegenes.org/mouse/release_M20.html) and input with a “-G” option. To visualize the sequencing tracks, BIGWIG files were generated from BAM files using deepTools version 3.2.0 [88], bamCoverage command with “-of bigwig -bs 1 --exactScaling --normalizeUsing CPM” parameters, and displayed in Integrative Genomics Viewer [89]. Read count table was produced using featureCounts version 1.6.3 with “-t exon -g gene_id --extraAttributes gene_name -M -s 0 -p -P -d 0 -D 500 -a gencode.vM20.annotation.gtf” parameters. RNA-Seq signals were visualized using Integrative Genomics Viewer (Version 2.11.3) (http://software.broadinstitute.org/software/igv/). The RNA-Seq data were deposited in the Gene Expression Omnibus under accession number GSE197494.

### Bioinformatics analysis

First, bioinformatics analysis was performed on the CLC Genomics Workbench software, which proposed to perform statistical analysis and create a heat map using quantification values of each gene. Second, the data were visualized and analyzed using some validated softwares. Scatter plot using values of TPM was created on Microsoft Excel 2016 (Microsoft, Redmond, WA, USA). PCA plot, Volcano plot, and MA plot were created on TCC-GUI (ref: 10.1186/s13104-019-4179-2), which is a R/Bioconductor package, using total count data of each gene. Gene ontology enrichment analysis was performed on DAVID Bioinformatics Resources 6.8 [90] as a web tool, applying gene lists of <0.05 as FDR *p* value. Venn diagram was created using VennDiagram as R package on R version4.1.1. DEGs were detected using the criteria FDR < 0.05 among tumor samples. Among kidney samples, DEGs were identified using the definition: FDR < 0.001 and log fold change (log FC) more than 2.

### TCGA data analysis

To evaluate the association between *SLC7A11*-positive macrophages and clinical outcome (overall survival), TCGA data was used through the Memorial Sloan Kettering Cancer Center cBioPortal for CANCER GENOMICS (http://www.cbioportal.org) [91, 92]. For crude survival analysis, overall survival of *PTPRC (CD45)*-high and *SLC7A11*-high expression patients was compared with that of *PTPRC (CD45)*-high and *SLC7A11*-low expression patients using the Cox proportional hazard model.

### Single-cell RNA-seq analysis

Published single-cell RNA-seq dataset was used through integrative web server short for single-cell RNA-seq data visualization and analyzation (scDVA) (http://panmyeloid.cancer-pku.cn/). This dataset included pan-cancer single-cell RNA expression of tumor-infiltrating myeloid cells [36]. All clusters and macrophage sub-clusters were already defined according to the previous report [36]. To detect *SLC7A11*-positive macrophages, we evaluated *SLC7A11* expression in macrophage clusters among kidney cancer, lung cancer, colon cancer, ovarian cancer, and pancreatic cancer specimens. For dimensionality reduction and visualization, UMAP was used.

### Scratch healing assay

RenCa (1 × 10^5^) cells were seeded on 6-well plates and incubated for 48 h at 37 °C until cells became confluent monolayer. To create a gap in the confluent monolayer of RenCa cells, each well was scraped in a straight line with a 200 μl pipet tip and washed with PBS. Next, the first image of the scratch was acquired and the gap area was measured using the ImageJ FIJI software (time 0). Sorted F4/80^low^Ly6C^low^ or F4/80^high^Ly6C^low^ macrophages (5 × 10^4^) were seeded on inserts of Transwell systems (3412, Corning, Glendale, AZ, USA) and these inserts were combined with the 6-well plates on which RenCa cells were seeded. After 24 h of incubation, each well was washed with PBS, and the second image of the scratch was obtained (time 24). The degree of gap closure was evaluated using ImageJ FIJI software. The percentage of gap closure area was calculated using the following formula: % closure area = (time 0–time 24)/time 0. To evaluate the effect of SSZ on macrophage function, sorted F4/80^low^Ly6C^low^ macrophages were cultured for 24 h in the culture media containing SSZ (300 μM) or PBS. After incubation, SSZ-treated F4/80^low^Ly6C^low^, PBS-treated F4/80^low^Ly6C^low^, or PBS-treated F4/80^high^Ly6C^low^ macrophages were co-cultured with RenCa cells in Transwell systems (3412, Corning, Glendale, AZ, USA) after creating a gap.

### MTS assay

RenCa cells (2 × 10^3^) were seeded on a 96-well plate. Twenty-four hours after incubation, SSZ (100, 200, 300, or 400 μM), PBS, or RPMI 1640 medium was added, and cells were incubated for 48 h. Sorted F4/80^low^Ly6C^low^ macrophages (1 × 10^4^) were seeded on 96-well plates and incubated overnight. SSZ (300 μM), PBS, or RPMI 1640 medium was added, and cells were incubated for 24 h or 48 h. Cells were washed using PBS and incubated for 1 h in RPMI 1640 medium containing 10% FBS and MTS reagent according to the manufacturer’s protocol (G3580, Promega, Madison, WI, USA). Cell viability was measured using a plate reader (PerkinElmer, Waltham, MA, USA). The percentage of cell viability was calculated by dividing the value of SSZ- or PBS-treated cells by the value of RPMI 1640-administered cells.

### LDH assay

RenCa cells (2 × 10^3^) were seeded on a 96-well plate. Twenty-four hours after incubation, SSZ (100, 200, 300, or 400 μM) or PBS was added, and cells were incubated for 48 h. Sorted F4/80^low^Ly6C^low^ macrophages (1 × 10^4^) were seeded on 96-well plates and incubated overnight. SSZ (300 μM) or PBS was added, and cells were incubated for 48 h. Cell toxicity was measured using the Cytotoxicity LDH Assay kit-WST (CK12, Dojindo Molecular Technologies, Inc., Kumamoto, Japan) following the manufacturer’s non-homogeneous protocol and a plate reader (PerkinElmer, Waltham, MA, USA). Cells treated with lysis buffer were used as the positive control. The percentage of cell toxicity was calculated by dividing the value of SSZ- or PBS-treated cells by the value of positive control cells.

### Cystine uptake assay

Sorted F4/80^low^Ly6C^low^ or F4/80^high^Ly6C^low^ macrophages (2 × 10^4^) were seeded on 96-well black plates and incubated for 48 h. Cells were washed using cystine-free DMEM (21013024, Thermo Fisher Scientific, Waltham, MA, USA) three times. Thereafter, SSZ (300 μM) or PBS was added and cells were incubated for 5 min. The uptake of cystine uptake was evaluated using the Cystine Uptake Assay kit (UP05, Dojindo Molecular Technologies, Inc., Kumamoto, Japan) according to the manufacturer’s protocol. The fluorescence intensities, which reflect cystine uptake activity [93], were measured using a plate reader (PerkinElmer, Waltham, MA, USA).

### Human data of kidney cancer patients

Forty-nine kidney cancer patients who received ICI therapy from October 2016 to April 2021 at the University of Tokyo hospital were included in this study. Patients were followed through July 2021. All patients were ≥20 years old. We excluded patients if they had incomplete eGFR profiles or received dialysis or kidney transplantation. CKD was defined as eGFR <60 ml/min/1.73 m^2^ at the time of ICI initiation. All demographic variables, including age, gender, ICI therapy regimen, Karnofsky performance status, hemoglobin level, calcium level, neutrophil level, and platelet level were collected from the time of ICI initiation by reviewing physician notes as well as laboratory results via the electronic medical record. We calculated the International Metastatic Renal Cell Carcinoma Database Consortium (IMDC) score according to the previous study [41]. Baseline characteristics were compared using the Student’s *t* test or the Mann–Whitney *U* test for continuous variables and the chi-square test for categorical variables. Patients who survived or were lost to follow-up during the study period or stopped ICI therapy due to other causes than disease progression were censored at the end of the follow-up period. To evaluate the effect of CKD on time to ICI failure, which was defined as the period from the date of ICI initiation to the date of ICI termination due to disease progression or death during ICI treatment, we performed multivariate Cox regression models and set age and IMDC score (0 or 1 vs. 2) as covariates.

This study was approved by the Institutional Review Board of the University of Tokyo hospital (approval number [G3511]). Written informed consent was provided by all patients. Tumor samples were surgically resected from each patient who received nephrectomy before ICI therapy at the University of Tokyo Hospital. This study was conducted following the principles of the Declaration of Helsinki.

### Statistical analyses

All data are presented as means ± SEM. Mann–Whitney *U* test was used to analyze continuous variables. For multiple comparisons, a Kruskal–Wallis test followed by a post hoc Dann–Bonferroni multiple comparisons test, if appropriate, was conducted. A *p* value <0.05 indicated significance. All statistical analyses were two-sided and were performed by using Stata version 15.1 (StataCorp LP, College Station, TX, USA). To draw bar graphs, GraphPad Prism 9 software (GraphPad Software Inc., San Diego, CA, USA) was used.

## Supplementary information

Supplementary Data Legends

Supplementary Tables

Extended Data Figures

Supplementary figure legends_clean

Supplementary Data 1

Supplementary Data 2

## Data Availability

FACS-sorted murine Ly6C^low^ macrophage RNA-seq data are available in the Gene Expression Omnibus (GEO) under accession number GSE197494. The Cancer Genomw Atlas data were used through the Memorial Sloan Kettering Cancer Center cBioPortal for CANCER GENOMICS (http://www.cbioportal.org). Published single-cell RNA-seq dataset was used through integrative web server short for single-cell RNA-seq data visualization and analyzation (scDVA) (http://panmyeloid.cancer-pku.cn/). Any additional data supporting the findings of this study are available from the corresponding author on reasonable request.
