# Supplementary material for: Effect of M2-like macrophages of the injured-kidney cortex on kidney cancer progression
Source: Cell Death Discov. 2022 Dec 5;8:480. doi: 10.1038/s41420-022-01255-3 (PMC9722672; doi:10.1038/s41420-022-01255-3)
Supplement: Supplementary file 1 — Supplementary Data Legends [file 41420_2022_1255_MOESM1_ESM.docx]

**Description of additional supplementary files**

**Supplementary Data 1**

**Description: List of all normalized gene expressions in RNA-seq.**

**Supplementary Data 2**

**Description: List of differentially expressed genes.**
