# Supplementary material for: Effect of M2-like macrophages of the injured-kidney cortex on kidney cancer progression
Source: Cell Death Discov. 2022 Dec 5;8:480. doi: 10.1038/s41420-022-01255-3 (PMC9722672; doi:10.1038/s41420-022-01255-3)
Supplement: Supplementary file 2 — Supplementary Tables [file 41420_2022_1255_MOESM2_ESM.docx]

**Supplementary Table 1. Patient characteristics stratified by CKD (eGFR<60).**

| Characteristics | Total (N=49) | CKD (N=36) | Non-CKD (N=13) | *P*-value |
| --- | --- | --- | --- | --- |
| Age, median (IQR) [year] | 68 (57–71) | 68 (58–71) | 69 (53–70) | 0.79 |
| ≥65, n (%) | 29 (52) | 21 (58.3) | 8 (61.5) | 0.84 |
| Gender (male), n (%) | 37 (75.5) | 28 (77.8) | 9 (69.2) | 0.54 |
| ICI regimen, n (%) |  |  |  | 0.57 |
| Nivolumab monotherapy | 23 (46.9) | 18 (50.0) | 5 (38.5) |  |
| Nivolumab + ipilimumab | 20 (40.8) | 15 (41.7) | 5 (38.5) |  |
| ICI + VEGF-TKI | 6 (12.3) | 3 (8.3) | 3 (23.0) |  |
| IMDC score, n (%) |  |  |  | 0.21 |
| 0 | 6 (12.2) | 4 (11.1) | 2 (15.4) |  |
| 1 | 32 (65.3) | 26 (72.2) | 6 (46.2) |  |
| 2 | 11 (22.5) | 6 (16.7) | 5 (38.5) |  |
| ICI stop due to PD, n (%) | 25 (51.0) | 19 (52.8) | 6 (46.2) | 0.68 |
| Progression-free ICI treatment duration, median (IQR) [days] | 112 (42–240) | 80 (37–203) | 152 (113–265) | 0.12 |

Abbreviations: CKD, chronic kidney disease; ICI, immune checkpoint inhibitor; IMDC, International Metastatic Renal Cell Carcinoma Database Consortium; IQR, interquartile range; min, minimum; PD, progressive disease.

**Supplementary Table 2. Factors related to the progression-free ICI therapy duration based on a Cox regression analysis (N=49).**

| Characteristics | Unadjusted hazard ratio (95% CI) | *P*-value | Adjusted hazard ratio (95% CI)^a^ | *P*-value |
| --- | --- | --- | --- | --- |
| Age |  |  |  |  |
| <65 years | Reference |  | Reference |  |
| ≥65 years | 0.74 (0.33–1.66) | 0.47 | 0.62 (0.27–1.42) | 0.26 |
| IMDC score |  |  |  |  |
| 0-1 | Reference |  | Reference |  |
| 2 | 2.73 (1.08–6.89) | 0.03 | 6.15 (1.78–21.3) | 0.004 |
| CKD |  |  |  |  |
| No | Reference |  | Reference |  |
| Yes | 1.42 (0.56–3.61) | 0.47 | 3.82 (1.10–13.3) | 0.035 |

^a^Adjusted for age, IMDC score, and CKD.

Abbreviations: CI, confidence interval; CKD, chronic kidney disease; ICI, immune checkpoint inhibitor; IMDC, International Metastatic Renal Cell Carcinoma Database Consortium.

**Supplementary Table 3. List of oligonucleotides used for qPCR in this study.**

| **Primer** | **Forward** | **Reverse** |
| --- | --- | --- |
| Mouse βactin | AAGATCAAGATCATTGCTCCTCCTG | AAACGCAGCTCAGTAACAGTCC |
| Mouse Arg1 | TCACCTGAGCTTTGATGTCG | TCACCTGAGCTTTGATGTCG |
| Mouse Vegfa | TTACTGCTGTACCTCCACCA | ACAGGACGGCTTGAAGATG |
| Mouse Il10 | AGGCGCTGTCATCGATTTCT | ATGGCCTTGTAGACACCTTGG |
| Mouse Il1β | CCTTCCAGGATGAGGACATGA | AACGTCACACACCAGCAGGTT |
| Mouse Il6 | ACAAAGCCAGAGTCCTTCAGAGAG | TTGGATGGTCTTGGTCCTTAGCCA |
| Mouse Tnfa | ACGTCGTAGCAAACCACCAA | ACGTCGTAGCAAACCACCAA |
| Mouse Slc7a11 | CTTTGTTGCCCTCTCCTGCTTC | CAGAGGAGTGTGCTTGTGGACA |
