# Supplementary material for: Effect of M2-like macrophages of the injured-kidney cortex on kidney cancer progression
Source: Cell Death Discov. 2022 Dec 5;8:480. doi: 10.1038/s41420-022-01255-3 (PMC9722672; doi:10.1038/s41420-022-01255-3)
Supplement: Supplementary file 4 — Supplementary figure legends_clean [file 41420_2022_1255_MOESM4_ESM.docx]

**Supplementary figure legends**

**Extended Data Figure 1. Kidney cancer progression on the AA-injected kidney is similarly enhanced with the increase of F4/80^low^Ly6C^low^ macrophages.**

**a,** AA was intraperitoneally administrated 14 days before euthanization. Representative Sirius red staining (left) and immunohistochemical analysis of F4/80 expression (right) in the kidney cortex are shown. Scale bars: 100 μm.

**b-f,** RenCa cells were orthotopically inoculated 14 days after AA or PBS administration, and mice were euthanized 20 days after inoculation. **b,** Picture of AA-tumors and PBS-tumors are shown. **c,** The tumor volume of each group is shown. (n=5 per group; two-sided Mann–Whitney *U* test) Percentages of F4/80^low^Ly6C^low^ cells **(d)**, F4/80^high^Ly6C^low^ cells **(e)**, and F4/80^low^Ly6C^high^ cells **(f)** among F4/80^+^ cells are shown. (n=5 per group; two-sided Mann–Whitney *U* test)

Abbreviations: AA, Aristolochic acid

**Extended Data Figure 2. Systemic factor of kidney injury does not affect cancer progression.**

**a,** Plasma creatinine level was measured 14 days after uIRI. (n=9 per group; two-sided Mann–Whitney *U* test) **b,** Plasma urea nitrogen level was measured 14 days after uIRI. (n=6 per group; two-sided Mann–Whitney *U* test) **c,** Picture of subcutaneously injected RenCa tumor after uIRI or sham operation is shown.

Abbreviations: uIRI, unilateral ischemia-reperfusion injury

**Extended Data Figure 3. Gene expression pattern of F4/80^low^Ly6C^low^ macrophages.**

To assess the characteristics of F4/80^low^Ly6C^low^ macrophages, gene expression pattern was evaluated using sorted F4/80^low^Ly6C^low^ and F4/80^high^Ly6C^low^ macrophages. **a-c,** The expression of M2 macrophage markers *Arg1* **(a)**, *Vegfa* **(b)**, and *Il10* **(c)** was evaluated in F4/80^low^Ly6C^low^ macrophages, compared to F4/80^high^Ly6C^low^ macrophages. (n=6 per group; two-sided Mann–Whitney *U* test) **d-f,** The expression levels of *Il1b* **(d)**, *Il6* **(e)**, and *Tnfa* **(f)** were compared between F4/80^low^Ly6C^low^ and F4/80^high^Ly6C^low^ macrophages (n=6 per group; two-sided Mann–Whitney *U* test)

**Extended Data Figure 4. Tumor-infiltrating T cell population is decreased in kidney cancer inoculated into AA-treated kidney subcapsule.**

**a-c,** Intratumor CD3^+^ **(a)**, CD4^+^ **(b)**, CD8^+^ T cell proportion in CD45^+^ cells **(c)** was quantitatively evaluated in kidney cancer inoculated into AA-treated kidney and kidney cancer inoculated into PBS-treated kidney subcapsule, as determined by flow cytometer. (n=4 per group; two-sided Mann–Whitney *U* test)

Abbreviations: AA, aristolochic acid

**Extended Data Figure 5. Tumor-infiltrating Treg population, T cell function, and exhausted T cell population are not different between uIRI-Can and Sham-Can.**

**a,** CD4^+^ FoxP3^+^ Treg cell proportion in CD4^+^ T cells in uIRI-Can and Sham-Can is shown. (n=6 per group; two-sided Mann–Whitney *U* test) **b-c,** Tumor-infiltrating IFNγ^+^ CD8^+^ T cell proportion **(b)** and TNFα^+^ CD8^+^ T cell proportion **(c)** among CD8^+^ T cells in uIRI-Can and Sham-Can are shown. (n=6 per group; two-sided Mann–Whitney *U* test) **d,** PD1^+^ Tim3^+^ CD8^+^ T cell proportion in CD8^+^ T cells in uIRI-Can and Sham-Can is shown. (n=6 per group; two-sided Mann–Whitney *U* test)

Abbreviations: uIRI, unilateral ischemia-reperfusion injury; uIRI-Can, kidney cancer inoculated into the kidney subcapsule after unilateral IRI; Sham-Can, kidney cancer inoculated into the kidney subcapsule after sham operation

**Extended Data Figure 6. F4/80^low^Ly6C^low^ macrophage proportion and gene expression of F4/80^low^Ly6C^low^ macrophages in uIRI kidney cortex.**

**a,** F4/80^low^Ly6C^low^ macrophage proportion in F4/80^+^ cells was evaluated in kidney cortex which was performed uIRI or sham operation 14 days before euthanization, as determined by flow cytometer. (n=4 per group; two-sided Mann–Whitney *U* test) **b,** To assess the characteristics of F4/80^low^Ly6C^low^ macrophages, gene expression was evaluated using F4/80^low^Ly6C^low^ and F4/80^high^Ly6C^low^ macrophages sorted from uIRI kidney cortex. F4/80^low^Ly6C^low^ macrophages expressed M2 macrophage markers *Arg1* and *Vegfa*, compared to F4/80^high^Ly6C^low^ macrophages. (n=3 per each F4/80^high^Ly6C^low^ macrophage group, n=4 per each F4/80^low^Ly6C^low^ macrophage group; two-sided Mann–Whitney *U* test)

Abbreviations: uIRI, unilateral ischemia-reperfusion injury

**Extended Data Figure 7. F4/80^low^Ly6C^low^ macrophage depletion from uIRI kidney with anti-CD8 or CD4 antibody.**

Anti-CD8 or anti-CD4 antibody or isotype control was administered every 6 days starting the day after RenCa cell inoculation into uIRI kidney subcapsule treated with CL or PBS after uIRI. Mice were euthanized 20 days after tumor inoculation. **a,** The experimental protocol is shown. **b,** Tumor-infiltrating CD8^+^ T cell proportion in CD45^+^ cells was quantitatively evaluated, as determined by flow cytometer; anti-CD8 antibody or isotype control administration to kidney cancer on PBS-treated uIRI kidney (left) (n=5 per group), anti-CD8 antibody or isotype control administration to kidney cancer on CL-treated uIRI kidney (right) (n=3 per isotype control group, n=5 per anti-CD8 antibody group; two-sided Mann–Whitney *U* test). **c-e,** The anti-CD4 antibody or isotype control was administered for tumor-bearing mice after CL or PBS treatment; anti-CD4 antibody or isotype control administration to kidney cancer on PBS-treated uIRI kidney (left) (n=4 per group), anti-CD4 antibody or isotype control administration to kidney cancer on CL-treated uIRI kidney (right) (n=4 per group; two-sided Mann–Whitney *U* test). **c,** Representative photos of each tumor are shown. **d,** Tumor volume is shown. **e,** The proportion of tumor-infiltrating CD4^+^ T cells in CD45^+^ cells was quantitatively evaluated, as determined by flow cytometer. (n=4 per each group)

Abbreviations: Lt. left side; uIRI, unilateral ischemia-reperfusion injury; CL, clodronate liposome; Ab, antibody

**Extended Data Figure 8. Confirmation of RNA-seq results.**

**a,** Representative gene tracks of RNA-seq signals around *Vegfa* in kidney samples and tumor samples. RNA-seq signals are visualized using Integrative Genomics Viewer (Version 2.11.3) (<http://software.broadinstitute.org/software/igv/>) **b,** qPCR confirmed that *Vegfa* was highly expressed in Ly6C^low^ macrophages sorted from uIRI kidney cortex (left) and uIRI-Can (right) compared to those from sham kidney cortex and Sham-Can. (n=3 per each kidney sample, n=4 per each tumor sample; two-sided Mann–Whitney *U* test) **c,** qPCR confirmed that *Slc7a11* was highly expressed in Ly6C^low^ macrophages sorted from uIRI kidney cortex (left) and uIRI-Can (right) compared to those from sham kidney cortex and Sham-Can. (n=3 per each kidney sample, n=4 per each tumor sample; two-sided Mann–Whitney *U* test) **d,** F4/80^low^Ly6C^low^ macrophage proportion in Ly6C^low^ cells was quantitatively evaluated in kidney sample (uIRI vs Sham kidney cortex) (left) and tumor sample (uIRI-Can vs Sham-Can) (right), as determined by cell sorter. (n=3 per each kidney sample, n=4 per each tumor sample; two-sided Mann–Whitney *U* test)

Abbreviations: uIRI, unilateral ischemia-reperfusion injury; uIRI-Can, kidney cancer inoculated into the kidney subcapsule after unilateral IRI; Sham-Can, kidney cancer inoculated into the kidney subcapsule after sham operation

**Extended Data Figure 9. The effects of sulfasalazine on RenCa cells and macrophages.**

**a-b,** RenCa cells were treated with SSZ (100, 200, 300, 400 μM) or PBS. (two-sided Mann–Whitney *U* test) **a,** Percent of cell viability was compared between the SSZ group and the PBS group using MTS assay. **b,** Percent of cell toxicity was compared between the SSZ group and the PBS group using LDH assay. Black circle represents the PBS group, and black triangle represents the SSZ group. **c,** Sorted F4/80^low^Ly6^low^ and F4/80^high^Ly6^low^ macrophages were treated with SSZ (300 μM) or PBS. Cystine uptake ability was compared between the SSZ group and the PBS group among F4/80^low^Ly6^low^ macrophages (left) and F4/80^high^Ly6^low^ macrophages (right). (two-sided Mann–Whitney *U* test) **d-e,** Sorted F4/80^low^Ly6^low^ macrophages were treated with SSZ (300 μM) or PBS. **d,** Percent of cell viability was compared between the SSZ group and the PBS group 24 or 48 h after SSZ or PBS treatment by MTS assay. Black circle represents the PBS group, and black triangle represents the SSZ group. (two-sided Mann–Whitney *U* test) **e,** Percent of cell toxicity was compared between the SSZ group and the PBS group by LDH assay. (two-sided Mann–Whitney *U* test) **f,** SSZ or PBS was administered every day starting 7 days after tumor inoculation into uIRI-operated kidney subcapsule. CD4^+^ FoxP3^+^ Treg cell proportion in CD4^+^ T cells was compared between the SSZ group and the PBS group. (n=5 per group; two-sided Mann–Whitney *U* test) **g,** RenCa cells were cocultured with F4/80^low^Ly6C^low^(M2-like), SSZ-treated F4/80^low^Ly6C^low^(M2-like+SSZ), or F4/80^high^Ly6C^low^(nonM2-like) macrophages in Transwell systems for 24 h after creating a gap in confluent monolayer of RenCa cells, and the degree of gap closure was evaluated. The protocol of this experiment is shown.

Abbreviations: uIRI, unilateral ischemia-reperfusion injury; uIRI-Can, kidney cancer inoculated into the kidney subcapsule after unilateral IRI; Sham-Can, kidney cancer inoculated into the kidney subcapsule after sham operation; SSZ, sulfasalazine

**Extended Data Figure 10. *SLC7A11* positive macrophages are identified in human kidney, lung, colon, ovarian, and pancreatic cancer.**

**a-b,** *SLC7A11*-positive macrophages were detected mainly in *GPNMB*-positive or *FN1*-positive macrophage sub-clusters among kidney cancer patients. **c,** Kaplan–Meier plot showing the overall survival of kidney cancer patients based on the expression level of *SLC7A11* in non-myeloid cells; blue line indicates *PTPRC*-low & *SLC7A11*-low patients, and red line indicates *PTPRC*-low & *SLC7A11*-high patients. The expression of *SLC7A11* in non–myeloid cells was not associated with prognosis among kidney cancer patients. Statistical analysis was performed using the two-sided log-rank test. HR was calculated using univariate the Cox regression analysis. **d-e,** *SLC7A11*-positive macrophages were also identified in lung, colon, ovarian, and pancreatic cancer patients, and these macrophages were mainly detected in *SPP1*-positive macrophage sub-clusters.
